# Dissecting the angiogenic mechanism of pachychoroid polypoidal choroidal vasculopathy

**DOI:** 10.1002/imt2.70176

**Published:** 2026-09-30

**Authors:** Xinyu Zhao, Tiantian Cheng, Wenfei Zhang, Xingwang Gu, Qing Zhao, Yaning Chen, Lihui Meng, Jiaqi Zhang, Bintao Qiu, Lili Li, Shiyu Cheng, Zicheng Wang, Chuting Wang, Yuelin Wang, Zuyi Yang, Zhengming Shi, Ye Guo, Shengzhi Liu, Youxin Chen

**Affiliations:** ^1^ Department of Ophthalmology Peking Union Medical College Hospital, Chinese Academy of Medical Sciences and Peking Union Medical College Beijing China; ^2^ Beijing Key Laboratory of Fundus Diseases Intelligent Diagnosis & Drug/Device Development and Translation Beijing China; ^3^ Key Laboratory of Ocular Fundus Diseases, Chinese Academy of Medical Sciences Beijing China; ^4^ Department of Ophthalmology, the First Affiliated Hospital, School of Medicine Zhejiang University Hangzhou Zhejiang China; ^5^ School of Pharmaceutical Sciences Capital Medical University Beijing China; ^6^ Beijing Area Major Laboratory of Peptide and Small Molecular Drugs Capital Medical University Beijing China; ^7^ Institute of Clinical Medicine National Infrastructure for Translational Medicine, Peking Union Medical College Hospital, Chinese Academy of Medical Sciences and Peking Union Medical College Beijing China; ^8^ State Key Laboratory of Common Mechanism Research of Major Diseases, Institute of Basic Medical Sciences Chinese Academy of Medical Sciences and Peking Union Medical College Beijing China; ^9^ Department of Ophthalmology Peking University Third Hospital Beijing China; ^10^ Department of Clinical Laboratory State Key Laboratory of Complex Severe and Rare Diseases, Peking Union Medical College Hospital, Chinese Academy of Medical Sciences and Peking Union Medical College Beijing China

**Keywords:** choroidal circulatory hypertension, disturbed shear stress, endothelin‐1, pachychoroid, polypoidal choroidal vasculopathy, vortex vein

## Abstract

Polypoidal choroidal vasculopathy (PCV) is a distinct variant of neovascular age‐related macular degeneration (AMD) and a major cause of vision loss in older adults, yet its pathogenesis remains poorly understood. Pachychoroid PCV is a subtype characterized by poor treatment response, minimal typical AMD features, and dilated choroidal vessels. Using a multicenter PCV cohort, we conducted plasma multi‐omics analyses and identified enrichment of fluid shear stress and atherosclerosis (FSS‐AS) signaling pathways in this subtype. The pachychoroid phenotype, together with FSS‐AS enrichment, implicated altered choroidal hemodynamics in its pathogenesis. By inducing choroidal circulatory hypertension (CCH) in mice, we recapitulated, for the first time, the hallmark ocular lesions of pachychoroid PCV. Single‐cell sequencing identified the cellular origin of these angiogenic lesions and implicated endothelin‐1 (EDN1) as a key effector. Alleviating choroidal hypertension, reducing EDN1 expression, or pharmacologically blocking the endothelin receptor A (EDNRA, the receptor for EDN1), prevented lesion formation. In vitro, disturbed fluid shear stress upregulated EDN1 in choroidal endothelial cells and promoted pathological remodeling and angiogenesis through EDNRA. Collectively, our findings define a pathogenic cascade wherein CCH induces abnormal hemodynamics (disrupted fluid shear stress) and drives angiogenesis via the EDN1/EDNRA axis, offering mechanistic insight and identifying a potential therapeutic target for pachychoroid PCV.

## Highlights

- A prognosis‐oriented classification for Pachy PCV was established using a multicenter dataset and corroborated by distinct plasma multi‐omics signatures.
- A novel mouse model induced by vortex vein ligation demonstrated that CCH is a critical driver of polypoidal lesions.
- Disturbed fluid shear stress and hypoxia disrupt choroidal endothelial homeostasis by upregulating the EDN1/EDNRA axis via FSS‐AS and HIF‐1 pathways.
- Targeting the EDN1/EDNRA signaling axis effectively attenuates pathological angiogenesis and vascular remodeling, highlighting a promising therapeutic strategy.

## Graphical abstract

A prognosis‐oriented classification system was established to distinguish Pachy PCV from Non‐pachy PCV using a large multicenter dataset. Comparative plasma multi‐omics profiling of Pachy PCV, Non‐pachy PCV, and normal controls revealed unique upregulation of the fluid shear stress and atherosclerosis (FSS‐AS) and HIF‐1 signaling pathways in Pachy PCV. Vortex vein‐ligation mouse models were then developed to demonstrate that choroidal circulatory hypertension (CCH) was a critical driver of polypoidal lesion formation in Pachy PCV. ScRNA‐seq analysis further identified *Edn1* as a key regulator of CCH‐induced PCV‐like lesion formation through the EDN1/EDNRA axis and the FSS‐AS and HIF‐1 pathways, which was further validated by both in vivo and in vitro experiments.

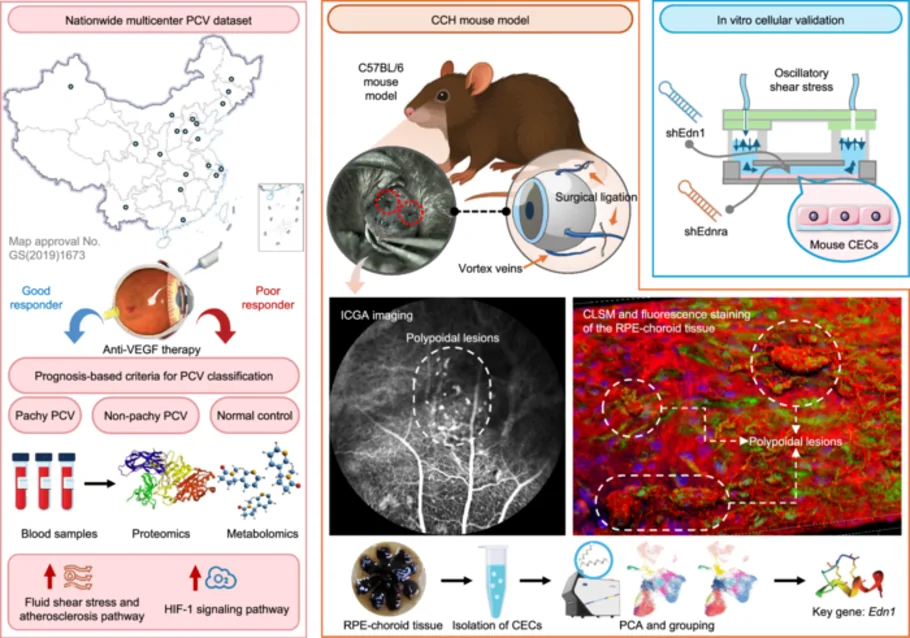

## INTRODUCTION

Polypoidal choroidal vasculopathy (PCV) is a choroidal vascular abnormality characterized by orange‐red polypoidal lesions and branching neovascular networks (BNNs) in the fundus [1, 2]. It is particularly prevalent in the Asian population, affecting an estimated 0.3% of the general Chinese population [3]. PCV has been reported to cause significant visual impairment and even blindness, primarily as a result of its severe ocular complications, including hemorrhagic pigment epithelial detachment (PED), massive subretinal hemorrhage (SRH), and breakthrough vitreous hemorrhage (VH) [4, 5]. However, the pathogenesis of PCV has remained unclear since its first description in 1990. Intravitreal injections of anti‐vascular endothelial growth factor (anti‐VEGF) drugs, currently regarded as the first‐line treatments for PCV, are costly and often yield suboptimal long‐term efficacy, thereby imposing a substantial burden on patients' families and global public health systems [6]. Therefore, advancing our understanding of the pathogenesis of PCV and identifying novel and effective therapeutic targets remain urgent priorities.

Recent studies have proposed categorizing PCV into pachychoroid PCV (Pachy PCV) and non‐pachychoroid PCV (Non‐pachy PCV) based on the bimodal distribution of subfoveal choroidal thickness (SFCT) [7]. Further evidence suggests that Pachy PCV, characterized by marked choroidal thickening, dilated and engorged vortex veins, increased maximum vascular diameter ratios, elevated choroidal vascularity indices, and notably increased choroidal vascular hyperpermeability (CVH) [8, 9], may represent a distinct clinical entity with potentially unique pathophysiological mechanisms. Additionally, patients with Pachy PCV tend to be younger and experience more severe hemorrhagic complications, which often require management with pars plana vitrectomy. Moreover, intraoperative rebleeding from polypoidal lesions is not unusual. Massive sand‐like, gray‐white hemorrhage may continuously extravasate from the subretinal space, posing extreme therapeutic challenges and necessitating the use of silicone oil or even perfluorocarbon liquid to stop the bleeding [10]. Based on these clinical observations, we hypothesized that choroidal circulatory hypertension (CCH), which contributes to the tendency to rupture and massive hemorrhage, might represent a key pathogenic driver in the development of Pachy PCV. However, existing studies have mainly focused on morphological characteristics in this respect, and the potential mechanisms underlying CCH in the development of Pachy PCV warrant further investigation.

In this study, we sought to elucidate whether and how CCH contributes to the development of Pachy PCV. We first examined the associations between CCH‐related characteristics and treatment responses to anti‐VEGF therapy in treatment‐naïve PCV patients using a prospective, multicenter cohort and proposed a new definition for Pachy PCV. Then, a Pachy PCV mouse model was established by inducing CCH through vortex vein ligation. Multi‐omics analyses, including proteomics, metabolomics, and single‐cell RNA‐sequencing (scRNA‐seq), were subsequently performed to identify key pathways and molecular drivers implicated in Pachy PCV. Finally, in vitro and in vivo experiments were conducted to validate these findings (Figure S1). We believe this integrated clinical‐experimental research provides novel insights and a theoretical basis for the pathological mechanism of Pachy PCV and illuminates promising avenues for its therapeutic development.

## RESULTS

### Classification of PCV based on the multicenter dataset

A total of 716 treatment‐naïve PCV eyes from 716 patients were recruited across 43 nationwide medical centers, and their clinical and imaging characteristics were analyzed (Figure 1, Figure S2A). No missing data were present for the outcome or variables included in the regression analyses. Compared to patients with a good response to anti‐VEGF therapy (good responder group, 276 eyes), the poor responder group (440 eyes) exhibited significantly younger age (66.86 ± 9.01 vs. 70.48 ± 9.70, *p* < 0.001), greater SFCT (362.15 ± 101.74 μm vs. 233.79 ± 77.42 μm, *p* < 0.001), higher prevalence of choroidal pachyvessels (82.7% vs. 16.3%, *p* < 0.001, see Materials and Methods for definition), and lower prevalence of age‐related macular degeneration (AMD)‐like features (18.0% vs. 72.1%, *p* < 0.001) (Table S1 and Figure S2B–G). Furthermore, a significantly higher proportion of patients in the poor responder group developed massive hemorrhage during follow‐up (31.4% vs. 17.4%, *p* < 0.001). Multivariable logistic regression using center‐clustered robust standard errors identified the presence of choroidal pachyvessels (OR, 7.989; 95% CI, 2.680–23.784, *p* < 0.001), massive hemorrhage (OR, 2.199; 95% CI, 1.410–3.431, *p* < 0.001) and SFCT (OR, 1.005; 95% CI, 1.001–1.008, *p* = 0.008) as significant predictors of poor response to anti‐VEGF therapy (Table S1). Mixed‐effects and leave‐one‐center‐out sensitivity analyses yielded consistent estimates, with no single center materially influencing the results (Tables S2 and S3). Center‐specific sample sizes and poor‐response rates are presented in Table S4. Furthermore, the findings were also robust to loss to follow‐up, with consistent effect estimates when all lost patients were alternatively assumed to be good responders or poor responders (Table S5).

**FIGURE 1:**
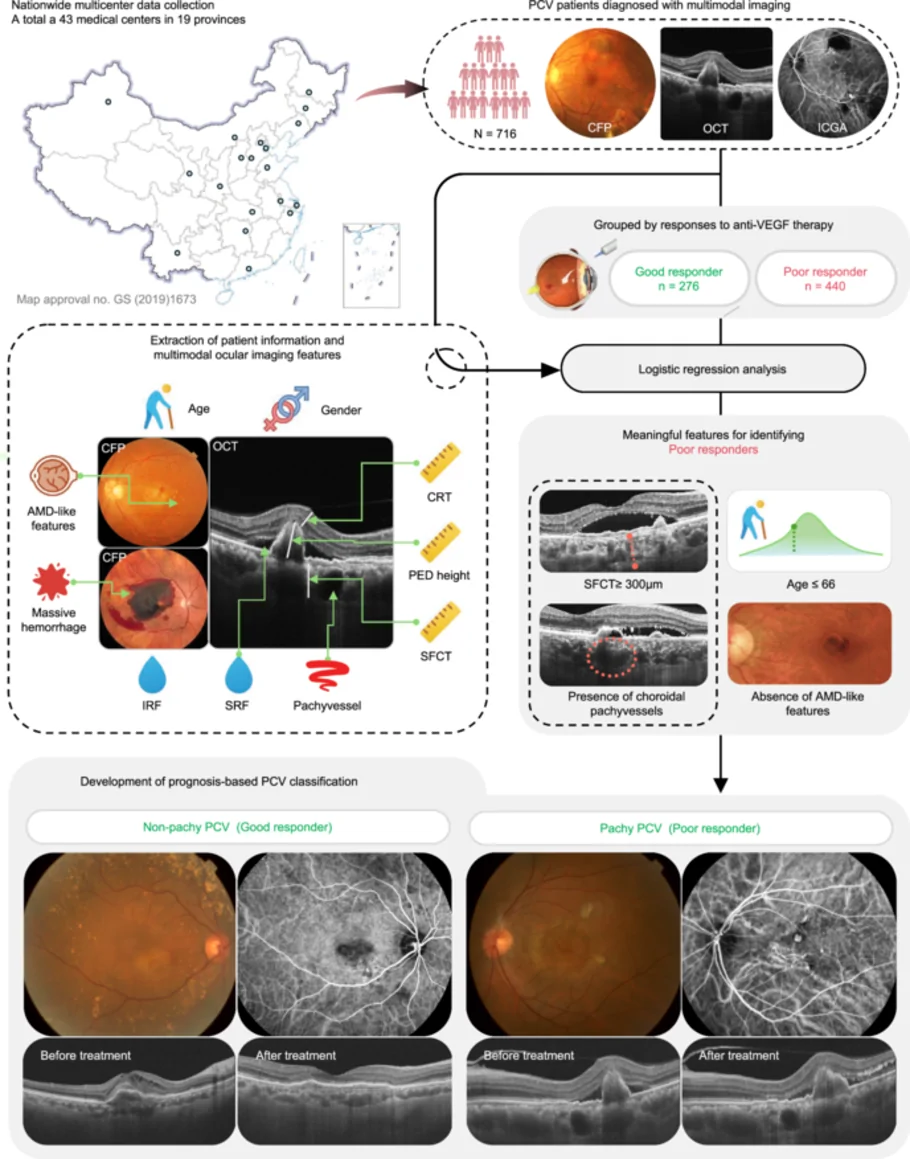
Development of prognosis‐based classification of PCV based on nationwide, multi‐center data. A total of 716 patients diagnosed with PCV were included from 43 medical centers across China. Patient demographics (e.g., age and sex) and multimodal ocular imaging features (e.g., SFCT measured on OCT and presence of massive hemorrhage on fundus photography), along with their responses to anti‐VEGF therapies, were analyzed. Among the 276 good responders and 440 poor responders, logistic regression analysis identified clinical features associated with treatment response and capable of distinguishing the two PCV subtypes. Based on these features, we developed a novel prognosis‐based classification system for PCV. Pachy PCV was defined by the presence of either both primary criteria or one primary criterion plus both secondary criteria. The primary criteria were (1) SFCT ≥ 300 µm and (2) the presence of choroidal pachyvessels. The secondary criteria were (1) age ≤ 66 years and (2) absence of AMD‐like features. Cases not meeting these criteria were classified as Non‐pachy PCV. Base map: Standard Map Service of the Ministry of Natural Resources of China. Map Approval No. GS(2019)1673. AMD, age‐related macular degeneration; CRT, central retinal thickness; IRF, intraretinal fluid; Non‐pachy, non‐pachychoroid; OCT, optical coherence tomography; PCV, polypoidal choroidal vasculopathy; PED, pigment epithelial detachment; Pachy, pachychoroid; SFCT, subfoveal choroidal thickness; SRF, subretinal fluid; VEGF, vascular endothelial growth factor.

Based on these findings, we proposed a novel classification system for PCV. Pachy PCV was defined as meeting either both primary criteria or one primary criterion together with both secondary criteria. Primary criteria included (1) SFCT ≥ 300 μm and (2) the presence of choroidal pachyvessels. Secondary criteria comprised (1) age ≤ 66 years and (2) absence of AMD‐like features. Cases not meeting these criteria were classified as Non‐pachy PCV. Although the optimal SFCT cutoff for predicting treatment response was 295.75 μm based on the Youden index (sensitivity + specificity − 1), with a sensitivity of 0.862 and a specificity of 0.832, a threshold of 300 μm was adopted for clinical applicability, given its comparable diagnostic performance. This set of criteria showed robust diagnostic performance, with high sensitivity (91.1%) and specificity (90.2%), allowing it to accurately differentiate Pachy PCV from Non‐pachy PCV (Table S6).

Following 1,000 bootstrap resamples for internal validation, the analysis demonstrated excellent discrimination for treatment response, with an apparent C‐index of 0.875 and an optimism‐corrected C‐index of 0.870. The calibration curve showed good overall agreement between the predicted probabilities and the observed outcomes, yielding a mean absolute error of 0.043. The optimism‐corrected calibration intercept and slope were −0.004 and 0.974, respectively, indicating minimal overall bias (Figure S2H). These validation results confirmed the high statistical robustness of the identified predictors, supporting their incorporation into the proposed classification criteria.

#### Proteomics and metabolomics revealed signaling pathways specific to Pachy PCV

Proteomic analyses of plasma samples from Pachy PCV patients (*n* = 40), Non‐pachy PCV patients (*n* = 31), and healthy controls (*n* = 35) identified 98 Pachy PCV‐specific proteins (Figure 2A, Figure S3A). Co‐expression analysis revealed that the predominant proteins of Pachy PCV (Module 3) were functionally enriched in signal transduction, response to stimulation, response to hypoxia, growth and proliferation regulation, inflammatory response, as well as cell adhesion and migration, which was significantly different from those in Non‐pachy PCV (Module 2) and healthy controls (Module 1) (Figure S4). Upregulation of pathways reflecting a transition from initial cellular response to active disease progression was observed in Pachy PCV (Figure 2B, Figure S5; full names of pathways are shown in Table S7). In addition to the VEGF, MAPK, Rap1, and PI3K‐Akt signaling pathways (Figure 2C, Figures S6, S7), two notable pathways, the fluid shear stress and atherosclerosis (FSS‐AS) pathway (proteins including NCF1, NCF2, PIK3CB, RAC1, RAC2, and RHOA) and the HIF‐1 signaling pathway (proteins including CAMK2G, EIF4E2, ENO1, PIK3CB, PRKCA, PRKCB, and RPS6) were identified (Figures S8, S9).

**FIGURE 2:**
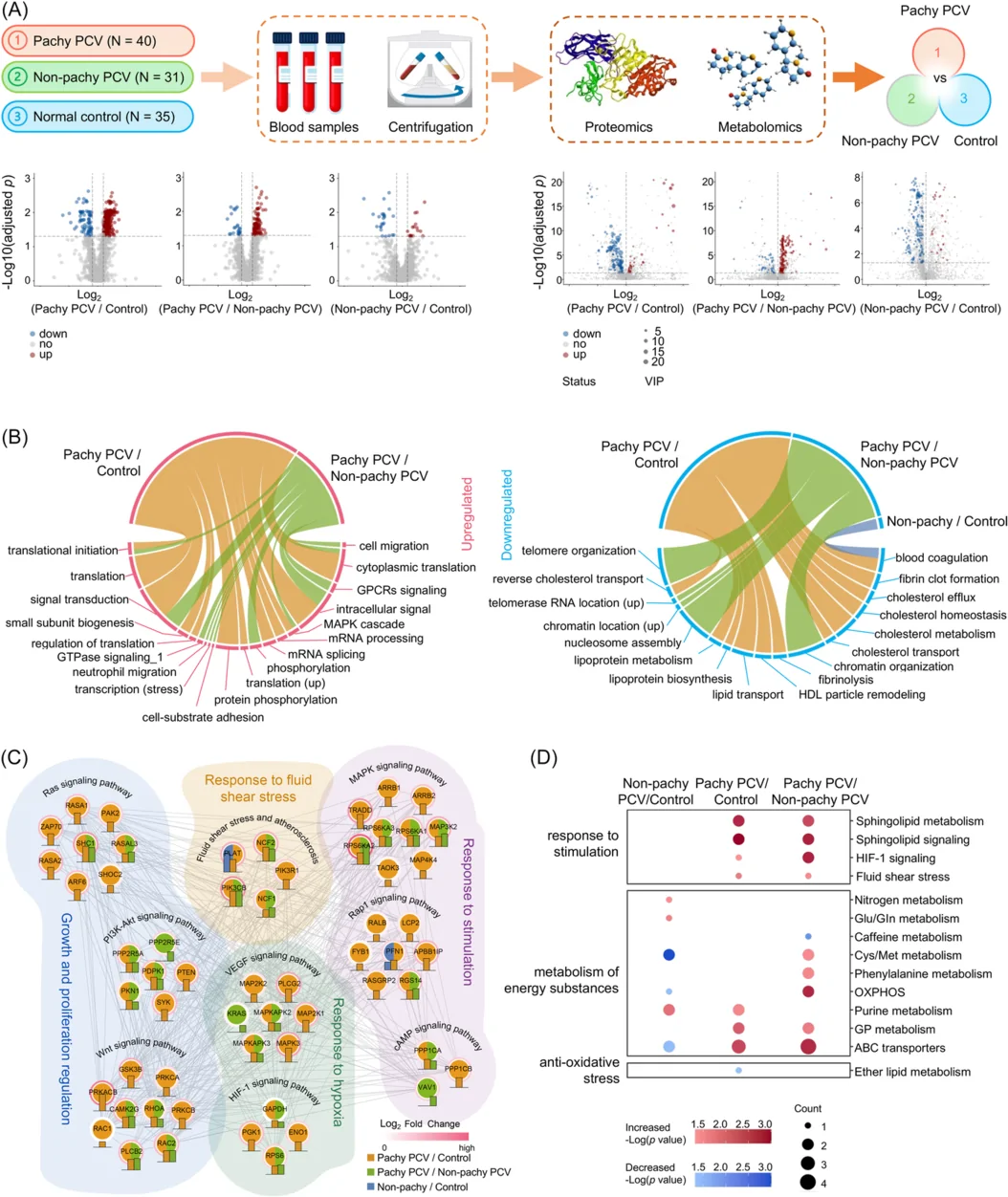
Plasma proteomic and metabolomic analyses reveal distinct signaling pathways associated with the pathogenesis of Pachy PCV and Non‐pachy PCV. (A) Differential proteins (left) and metabolites (right) identified among the Pachy PCV (*n* = 40), Non‐pachy PCV (*n* = 31), and control (*n* = 35) groups. (B) Chord diagram showing GO enrichment analysis of differential proteins among the three groups. (C) Network diagram showing KEGG pathway enrichment analysis of differential proteins among the three groups. (D) Grouped bubble plot showing KEGG pathway enrichment of differential metabolites among the three groups. GO, Gene Ontology; KEGG, Kyoto Encyclopedia of Genes and Genomes.

Metabolomic analysis identified 37 Pachy PCV‐specific metabolites (Figure 2A, Figure S3A). Consistent with proteomics, the predominant metabolites of Pachy PCV were enriched in pathways related to responses to stimulation and energy metabolism. In addition, the FSS‐AS pathway, including metabolites like peroxynitrite, and the HIF‐1 pathway, including metabolites like pyruvic acid and Fe^2+^, were also detected (Figure 2D, Figures S10, S11). Correlation analysis between the proteomic and metabolomic data (Figure S3B) indicated that the FSS‐AS and HIF‐1 pathways were consistently upregulated, positively correlating with pathways of stimulation response and energy metabolism. Univariable analysis showed that the differentially expressed plasma proteins and metabolites had high discriminatory power in distinguishing Pachy PCV from Non‐pachy PCV (Figure S3C).

### Mouse model establishment and imaging assessment

By ligating two of the four vortex veins in healthy C57BL/6 mice, we successfully induced Pachy PCV‐like ocular changes (Figure 3A–C, Figure S12), including vortex vein expansion and polypoidal lesions on indocyanine green angiography (ICGA). Vortex vein expansion refers to an increase in the vascular width of vortex veins; polypoidal lesions refer to focal, nodular hyperfluorescent foci on ICGA. On optical coherence tomography (OCT) imaging, we also observed double‐layer sign, thumb‐like protrusions, serous PED, and choroidal thickening, which are all typical features found in human PCV. Furthermore, H&E staining revealed choroidal vessel dilation and neovasculature originating from the choroidal vessels (Figure 3D). Fluorescence staining and confocal laser scanning microscopy (CLSM) of the retinal pigment epithelium (RPE)‐choroid complexes harvested from the mouse eyes further confirmed the existence of polypoidal lesions and BNN‐like structures (Figure 3E, Figure S13). The latter represented low‐lying, irregular, interconnected networks around the polypoidal lesions. Double staining with Isolectin‐B4 (IB4, red fluorescence for mCECs) and P‐Selectin (SELP, green fluorescence for the abnormal structures) in these areas further suggested their vascular nature.

**FIGURE 3:**
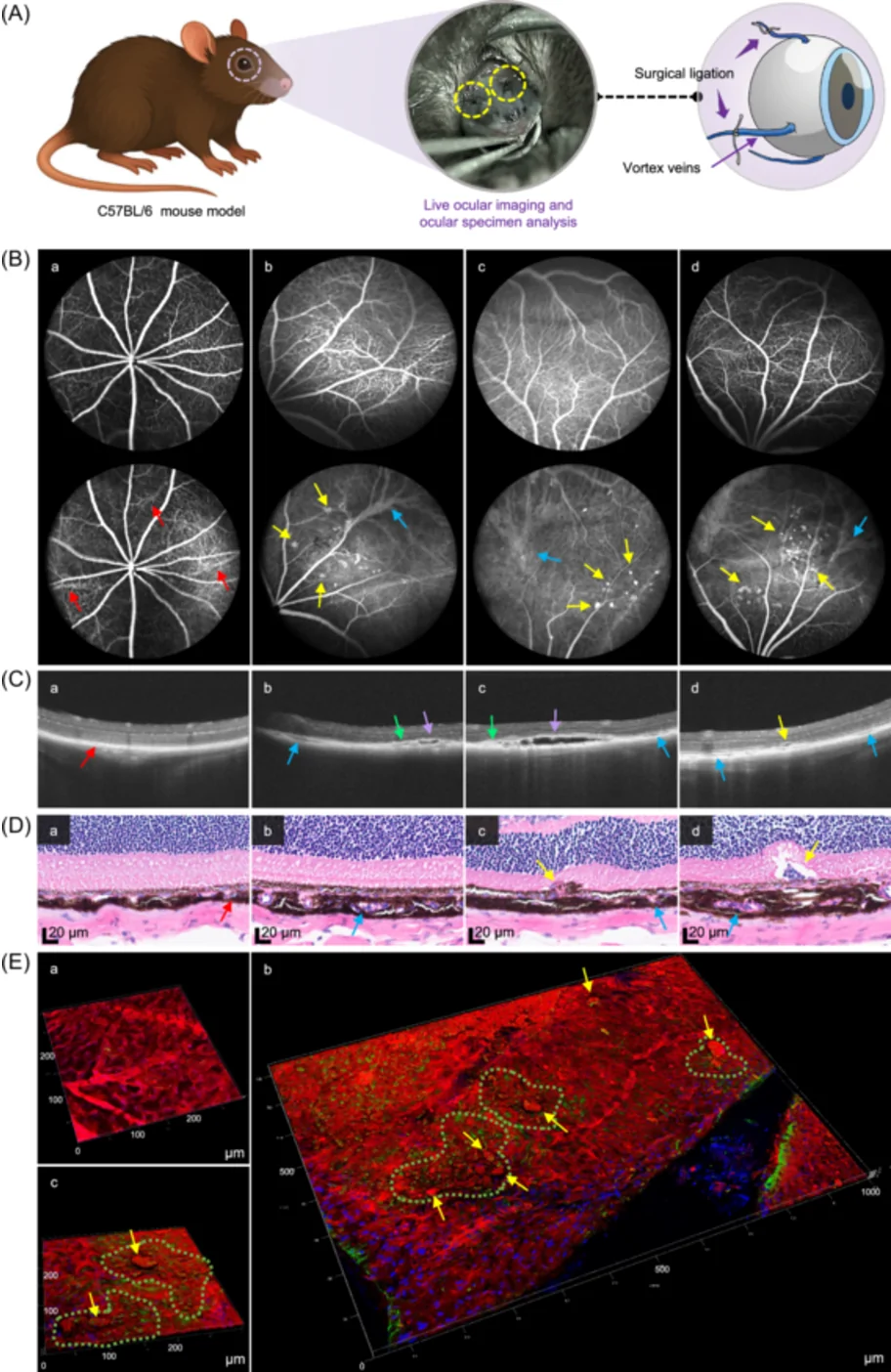
Establishment and assessment of PCV mouse model via vortex vein ligation. (A) Two vortex veins in the superior temporal and superior nasal quadrants of the C57BL/6 mouse were ligated for 30 days, followed by in vivo ocular imaging to assess the development of PCV‐like lesions. (B) ICGA images showed normal vortex veins (red arrows) in healthy control mice (subpanel a), whereas ligated mice (subpanels b–d) exhibited characteristic polypoidal lesions (yellow arrows) and dilated vortex vein (blue arrows). These lesions were not detected by FFA images. (C) OCT images showed a normal thin choroid (red arrows) in healthy control mice (subpanel a), whereas ligated mice (subpanels b–d) exhibited PCV‐like features, including the double‐layer sign (green arrows), thumb‐like protrusions (yellow arrows), dilated large choroidal vessels (blue arrows), and serous PED (purple arrows). (D) H&E staining showed that compared with non‐dilated choroidal vessels (red arrow in subgroup a) in healthy controls, whereas ligated mice (subpanels b–d) exhibited significant dilation of choroidal vessels (blue arrows). Abnormal vascular protrusions continuous with the choroidal vessels were observed beneath the RPE (yellow arrows, subpanel c), in some cases, penetrating the RPE layer (yellow arrows, subpanel d), consistent with polypoidal lesions extending into the subretinal space. (E) Confocal laser scanning of fluorescence‐stained RPE‐choroidal tissue showed Isolectin‐B4^+^ for mCECs (red) with a normal vascular morphology in control mice (subpanel a). In ligated mice (subpanels b and c), nodular lesions (yellow arrows) and BNN‐like structures (green dashed circles) were observed. The BNN‐like structures consisted of low‐lying, irregular, interconnected vascular networks around the polypoidal lesions. These lesions showed colocalization of double‐stained with Isolectin‐B4 (red) and P‐selectin (green), indicating polypoidal lesion‐ and BNN‐related choroidal neovascularization. Higher‐magnification images of small polypoidal lesions are shown in Figure S13. Nuclei were counterstained with DAPI (blue). BNN, branching neovascular network; DAPI, 4',6‐diamidino‐2‐phenylindole; FFA, fundus fluorescein angiography; H&E, hematoxylin and eosin; ICGA, indocyanine green angiography; mCEC, mouse choroidal endothelial cell; RPE, retinal pigment epithelium.

### Identification of unique vascular endothelial cells in pachy PCV mouse model via scRNA‐seq

In the scRNA‐seq analysis of RPE‐choroid‐sclera complexes harvested from the Pachy PCV mouse model, four major types of endothelial cells (ECs) were identified and subclassified based on cluster‐specific gene expression: arterial, venous, capillary, and lymphatic (Figure 4A–D, Figure S14A; representative annotated genes are shown in Table S8). In addition, a distinct EC subtype was identified and termed polypoidal lesion and BNN‐related choroidal neovascularization (PB‐CNV, Figure 4A,B and Table S8). These unique ECs expressed collagen‐related genes (e.g., *Col4a1*, *Col4a2*) and activation‐related genes (e.g., *Sparc*, *Sparcl1*).

**FIGURE 4:**
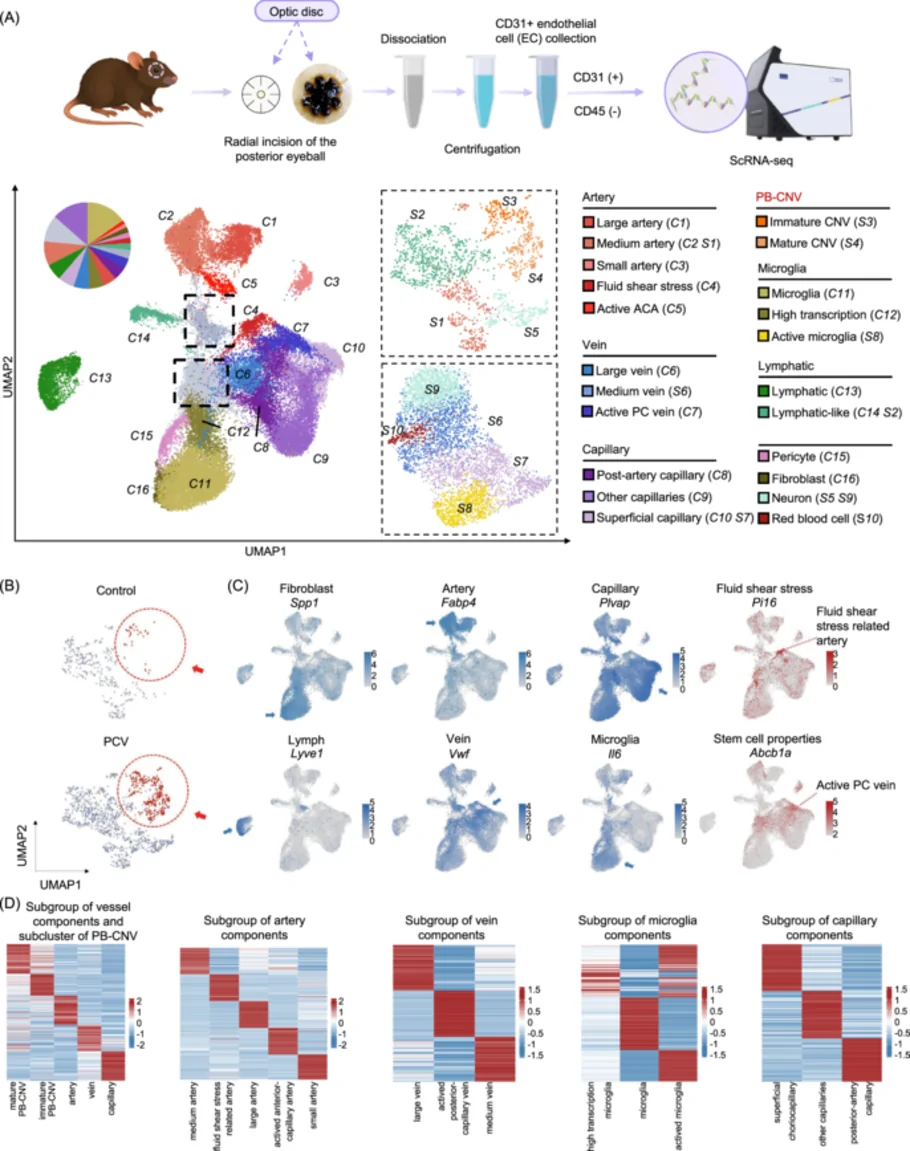
Cell annotation and identification of CNV components in RPE‐choroidal tissue from the mouse model by scRNA‐seq analysis. (A) RPE‐choroidal tissues were collected from ligated mice after sacrifice. Following radial incisions around the optic disc and endothelial cell isolation, scRNA‐seq was conducted. Transcriptome‐based dimensionality reduction, cell clustering and annotation, and subgroup‐specific distributions were shown within the frame. A novel endothelial cell population, termed PB‐CNV, was identified specifically in ligated mice. (B) Parallel comparison of cell populations between the ligation and control groups. CNV components were highlighted by red circles and arrows. (C) UMAP plots showing the distribution of marker gene expression. (D) Heatmap showing the expression levels of top 50 genes across different cell types. ACA, anterior‐capillary‐artery; CNV, choroidal neovascularization; PC, posterior‐capillary; scRNA‐seq, single‐cell RNA‐sequencing; UMAP, uniform manifold approximation and projection.

Pseudotime trajectory analysis suggested that PB‐CNV progenitor cells may arise from active posterior‐capillary (PC) vein cells and subsequently progress toward immature and mature PB‐CNV states (Figure 5A,B). Along the pseudotime axis, genes associated with stress injury (*Nupr1, Hmox1*), cytoskeleton‐related genes (*Pdlim1*), inflammation (*Sele* and *Selp*), and fibrosis (*Serpine1*) were gradually upregulated (Figure S14B). SELP was then selected as the biomarker for PB‐CNV, with IB4 as another biomarker for mouse choroidal ECs (mCECs). These markers were used for double EC staining of PB‐CNV in the above fluorescence staining and CLSM procedure.

**FIGURE 5:**
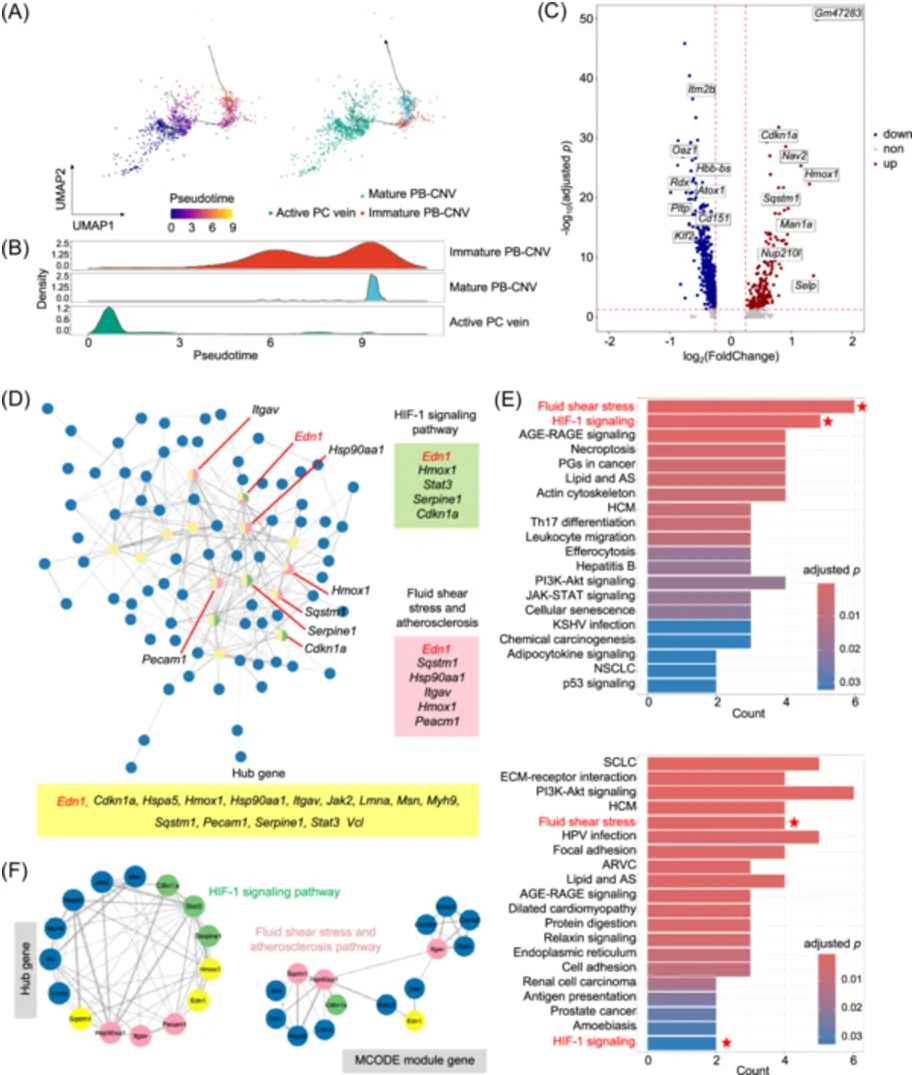
Inference of the cellular origin of PB‐CNV and identification of candidate target genes in PB‐CNV progenitor cells. (A) Pseudotime trajectory analysis of the cellular origin of PB‐CNV in the ligation group. (B) Density distribution of PB‐CNV progenitor cells and subtypes along the pseudotime trajectory. (C) Differentially expressed genes between ligation and control groups in the active posterior‐capillary vein (active PC vein, progenitor cells of PB‐CNV). (D) PPI network of upregulated differentially expressed genes in the active PC vein. The top 15 candidate hub genes were highlighted in yellow. Additional panels showed the distribution within the PPI network enriched in the fluid shear stress (pink) pathway and HIF‐1 signaling pathway (green). (E) KEGG enrichment analyses of the hub genes (upper) and MCODE module genes (lower). Key pathways were highlighted, indicated by stars. (F) PPI networks constructed from the hub genes and MCODE module genes. Genes associated with different pathways were color‐coded: pink, fluid shear stress‐related pathway; green, HIF‐1 signaling pathway; yellow, both pathways; and blue, other genes. *Edn1* (Endothelin‐1) was identified as a potential link between the fluid shear stress‐related and HIF‐1 signaling pathways. HIF‐1, hypoxia inducible factor‐1; MCODE, molecular complex detection; PPI, protein–protein interaction.

Gene co‐regulation module analysis and Gene Ontology (GO) enrichment revealed that the active PC vein (Modules 3 and 5) was involved in cell migration, proliferation, differentiation, extracellular matrix organization, and injury repair, representing the initiation of PB‐CNV development (Figure 5C, Figures S15–18A).

### Identification of *Edn1* as a key pathogenic target gene in the active PC vein

A protein–protein interaction (PPI) network of upregulated genes highlighted several activated pathways, with the most notable changes occurring in the FSS‐AS and HIF‐1 signaling pathways. Fifteen hub genes were preliminarily identified based on topological parameters, including *Edn1* (endothelin‐1), *Cdkn1a*, *Hspa5*, etc. (Figure 5D, Figure S18B). Consistently, Kyoto Encyclopedia of Genes and Genomes (KEGG) enrichment analysis of the hub genes, together with molecular complex detection (MCODE) subnetwork analysis, further demonstrated significant enrichment of both the FSS‐AS and HIF‐1 signaling pathways (Figure 5E). Notably, *Edn1* emerged as a potential molecular link between the FSS‐AS and HIF‐1 signaling pathways, with *Cdkn1a*, *Itgav*, *Hsp90aa1*, and *Sqstm1* also identified as key contributors within this regulatory network (Figure 5F, Figure S18C).

### In vitro effects of CCH‐induced oscillatory shear stress mediated via the EDN1/EDNRA axis

Based on the characteristic CCH observed clinically and recapitulated in the vortex vein ligation mouse model, together with the uniquely upregulated FSS‐AS pathway identified in Pachy PCV, oscillatory shear stress (OSS) was proposed and applied to simulate CCH‐induced abnormal blood flow and to evaluate its impact on mCECs in vitro. Immunofluorescence staining of cytoskeleton protein F‐actin and the endothelial cell adhesion molecule VE‐cadherin was performed to assess OSS‐induced cellular structural changes (Figure 6A). The qPCR analysis of 38 candidate genes exhibiting marked changes in expression indeed identified EDN1 as a key regulator mediating OSS‐induced cellular responses (Figure 6B). Live‐cell imaging revealed that under OSS, individual mCECs exhibited more rapid outward‐spreading, random walk‐like migratory trajectories compared with laminar shear stress controls, an effect that was markedly attenuated following *Edn1* knockdown using specific shRNA (Figure 6C). In addition, *Edn1* knockdown significantly suppressed OSS‐induced cellular phenotypes, including reduced proliferation, enhanced migration and invasion, and increased endothelial tube formation (Figure 6D,E, Figure S19A,B). These functional changes were accompanied by increased expression of c‐Casp3 (a marker of apoptosis), ANG‐1 (a vascular growth factor), MMP9 (an extracellular‐matrix‐remodeling protease), and IL‐6 (an inflammatory factor), and decreased expression of ZO‐1 (a tight‐junction protein), as demonstrated by Western blot analysis (Figure 6F). Conversely, re‐expression of *Edn1* via lentiviral overexpression in *Edn1* knockdown mCECs restored OSS‐induced cellular effects, including alterations in proliferation, migration and invasion, oxidative stress levels, and functional protein expression profiles (Figure 6G–K, Figure S19C). Co‐immunoprecipitation analysis further demonstrated a physical interaction between EDN1 and its receptor, endothelin receptor A (EDNRA), in mCECs (Figure 6L). Consistently, shRNA‐mediated knockdown of *Ednra* phenocopied the effects of *Edn1* knockdown, effectively abolishing OSS‐mediated cellular responses (Figure 6M–Q, Figure S19D).

**FIGURE 6:**
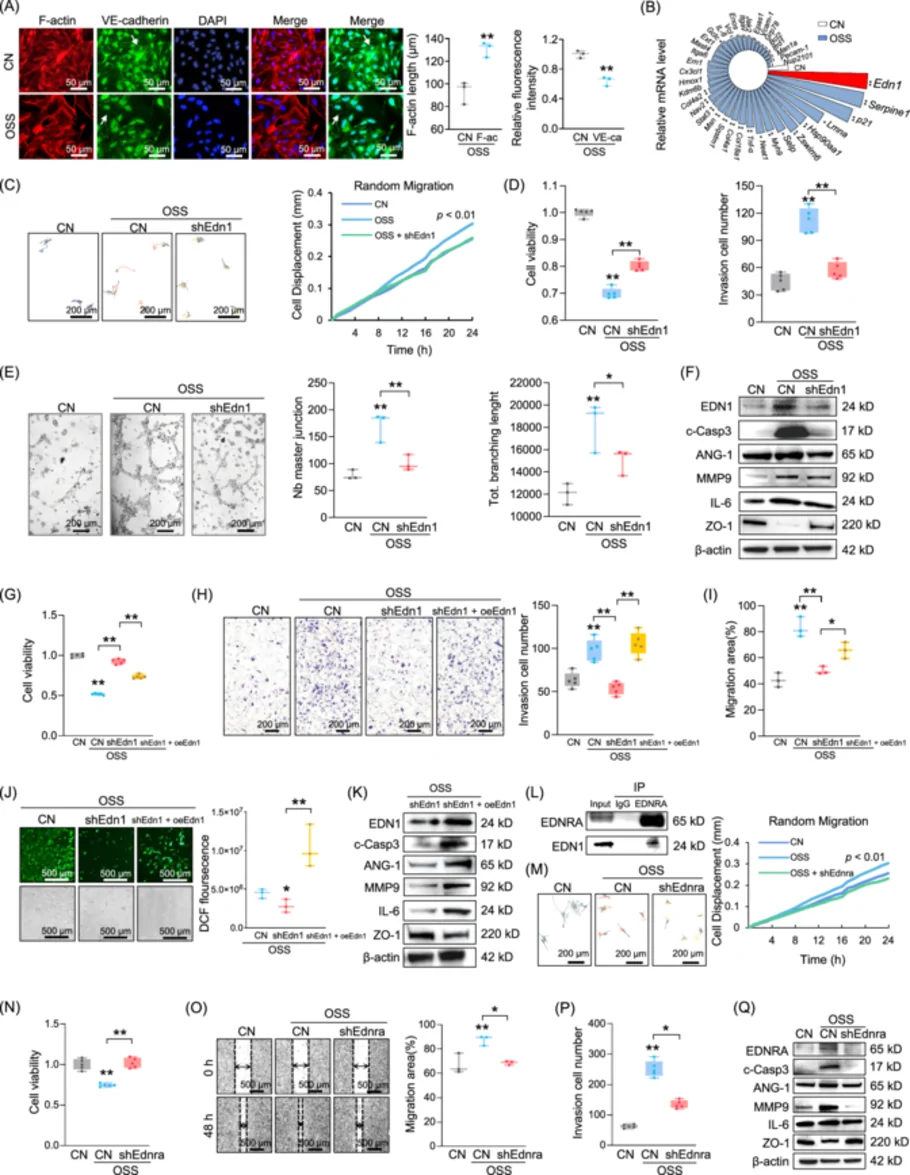
Effects of OSS on mCECs following *Edn1* knockdown and re‐expression and *Ednra* knockdown. (A) Representative immunofluorescence images of F‐actin and VE‐cadherin. F‐actin length and VE‐cadherin fluorescence intensity were quantified using ImageJ (version 1.54p). *n* = 3 independent biological replicates per group. (B) Potential targets for single‐cell sequencing based on qRT‐PCR screening. *n* = 3 independent biological replicates per group. (C) Representative trajectories of mCECs under laminar flows shear stress and oscillatory flow shear stress with and without *Edn1* shRNA. The change in displacement in 24 h was determined for each experiment and used as a single data point (*n* = 3 independent biological replicates per group). (D) CCK8‐based proliferation and transwell‐based invasion assays assessing the effects of OSS and *Edn1* knockdown. *Edn1* silencing significantly attenuated the OSS‐induced reduction in proliferation and increase in invasion of mCECs. *n* = 5 independent biological replicates. (E) Tube formation of mCECs under OSS with or without *Edn1* knockdown. OSS promoted tube formation, whereas *Edn1* knockdown attenuated this effect. The number of master junctions and total branching length were quantified using ImageJ, *n* = 3 independent biological replicates. (F) Representative Western blots showing the expression of EDN1, c‐Casp3, ANG‐1, MMP9, IL‐6, and ZO‐1 in response to OSS with and without *Edn1* shRNA. *n* = 3 independent biological replicates. (G) CCK8 assays of mCEC proliferation under OSS following *Edn1* re‐expression after *Edn1* knockdown. *n* = 5 independent biological replicates. Transwell invasion (H) and scratch migration (I) assays showing that the OSS‐induced invasive and migratory responses of mCEC cells were attenuated by *Edn1* knockdown and restored by *Edn1* re‐expression. *n* = 5 independent biological replicates per group for the Transwell invasion assay and *n* = 3 for the scratch migration assay. (J) Representative fluorescence images of ROS production in mCECs exposed to OSS following *Edn1* knockdown and/or re‐expression. Intracellular ROS levels were assessed by DCF fluorescence. *n* = 3 independent biological replicates per group. (K) Expression of EDN1, c‐Casp3, ANG‐1, MMP9, IL‐6, and ZO‐1 following *Edn1* knockdown and subsequent *Edn1* re‐expression. *n* = 3 independent biological replicates per group. (L) Co‐immunoprecipitation showing the interaction between EDN1 and EDNRA in mCECs protein extracts. *n* = 3 independent biological replicates. (M) Representative trajectories of mCECs exposed to OSS with or without *Ednra* knockdown. The change in displacement over 24 h was calculated for each independent experiment. *n* = 3 independent biological replicates per group. (N) CCK8 assay assessing the effects of OSS and *Ednra* knockdown on mCEC proliferation. *Ednra* silencing significantly attenuated the OSS‐induced reduction in proliferation. *n* = 5 independent biological replicates per group. Scratch migration (O) and Transwell invasion (P) assays showing that OSS increased the migratory and invasive capacities of mCECs and that these effects were attenuated by Ednra knockdown. *n* = 3 independent biological experiments for the scratch migration assay and *n* = 5 for the transwell invasion assay. (Q) *Ednra* silencing reduced the expression of EDNRA, c‐Casp3, ANG‐1, MMP9, and IL‐6 and increased ZO‐1 expression. *n* = 3 independent biological replicates per group. OSS, oscillatory shear stress (±6 dyn/cm^2^, 12 h); CN, control (laminar shear stress at 6 dyn/cm^2^); shEdn1, *Edn1* shRNA, oeEdn1, over‐expressed *Edn1*; shEdnra, *Ednra* shRNA; qRT‐PCR, quantitative reverse transcription polymerase chain reaction; CCK‐8, Cell Counting Kit‐8; DCF, dichlorofluorescein; IP, immunoprecipitation; IgG, immunoglobulin G; ROS, reactive oxygen species. Data are presented as mean ± SD from n independent biological replicates. For two‐group comparisons, statistical significance was assessed by two‐tailed unpaired Student's *t*‐test; for multiple groups, by one‐way ANOVA followed by Tukey's multiple comparisons test. Independent biological replicates: separate cell‐culture experiments per condition. The single and double asterisks indicate *p* < 0.05 and *p* < 0.01, respectively.

### In vivo validation of the role of CCH and EDN1/EDNRA axis

To further validate the in vivo role of EDN1/EDNRA, we then constructed comparative vortex vein ligation mouse models with *Edn1* ^*+/*^^−^ or the administration of an EDNRA antagonist. A vehicle control group for EDNRA antagonist gavage was also constructed to exclude the potential effect of the drug solvent itself. Because vortex vein ligation induced the CCH status, one additional model with vortex vein ligation and simultaneous scleral fenestration to release the choroidal hypertension was also constructed for comparison (Figure 7A–C). Among all experimental groups, mice subjected to vortex vein ligation alone exhibited the highest proportion of polypoidal lesions on ICGA (11 out of 23 eyes [47.8%]), with an average of 5.96 polypoidal lesions per eye, as well as the greatest increases in the maximal vortex vein diameter (1536.6 ± 119.1 µm) and choroidal thickness (58.20 ± 3.56 µm) compared to other groups (all *p* < 0.05) (Figure 7D–H). In contrast, simultaneous scleral fenestration, EDNRA antagonist, and *Edn1* ^*+/*^^−^ group each attenuated polypoidal lesion formation, vortex vein dilation, and choroidal thickening compared with the ligation‐only group (*p* < 0.05). The vehicle control group with vortex vein ligation with drug solvent without EDNRA antagonist showed similar results as the ligation‐only group (detailed in Figure S20). These findings remained robust after accounting for the non‐independence of fellow eyes at the mouse level using mixed‐effects or cluster‐adjusted models. Significant overall between‐group differences persisted for polyp occurrence, polyp number, maximal vortex vein diameter, and choroidal thickness after multiplicity adjustment (adjusted *p* < 0.05). Notably, vortex ligation was performed unilaterally in *Edn1* ^*+/*^^−^ mice, but no polypoidal lesion developed in either eye. This occurred despite the ligated eyes exhibiting increased maximal vortex vein diameter and choroidal thickness relative to the contralateral non‐ligated eyes.

**FIGURE 7:**
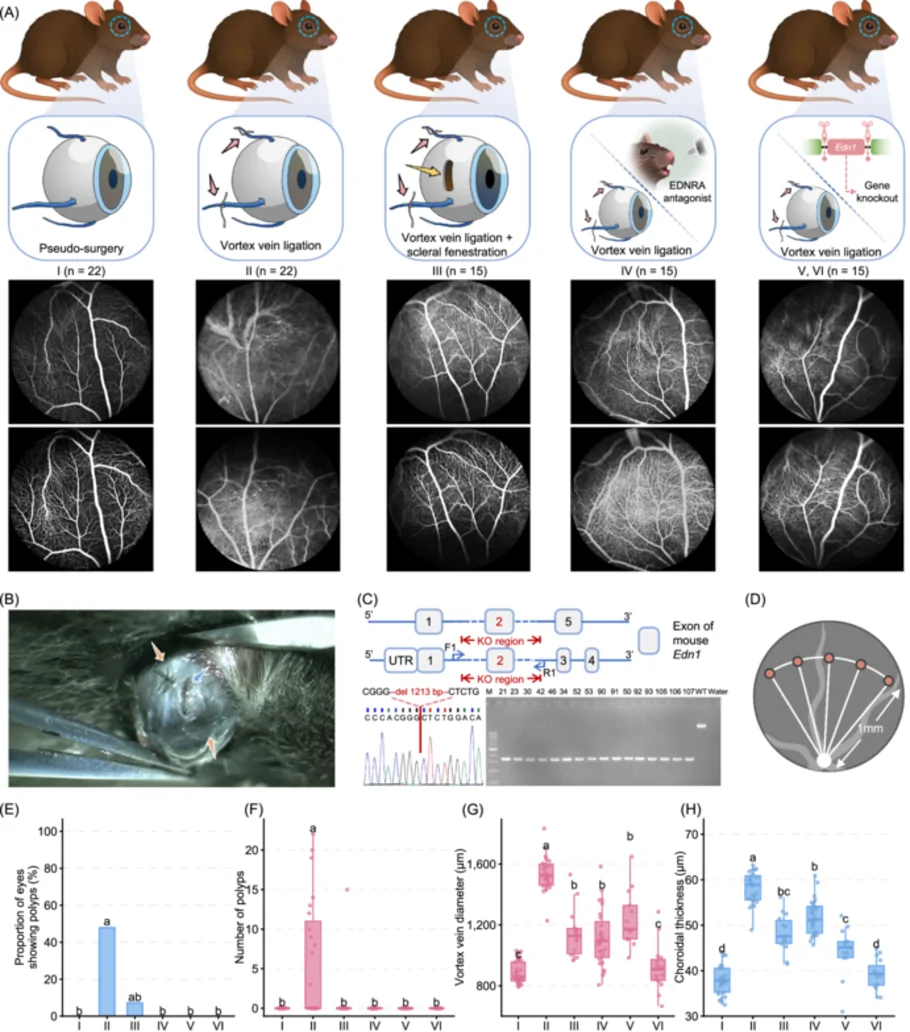
In vivo validation of the role of *Edn1* and CCH in the vortex vein ligation mouse model. (A) In addition to the baseline pseudo‐surgery (I) and vortex vein ligation (II) groups of C57BL/6 mice, four additional experimental groups were established: vortex vein ligation with scleral fenestration adjacent to the ligation site (III); vortex vein ligation with daily administration of an EDNRA antagonist (IV); systemic *Edn1* ^*+/*^^−^ with vortex vein ligation in one eye (V); systemic *Edn1* ^*+/*^^−^ only in the fellow eye (VI). Among the six subgroups, mice subjected to vortex vein ligation alone (II) developed numerous polypoidal lesions and marked vortex vein dilation on ICGA. (B) Representative photograph showing surgical ligation of vortex veins (pink arrows) and adjacent scleral fenestration (blue arrow). (C) Schematic of the heterozygous *Edn1* knockout (*Edn1* ^*+/*^^−^) strategy and validation by agarose gel electrophoresis. PCR amplification of tail genomic DNA showed that CRISPR‐Cas9‐mediated deletion of a 1213 bp fragment spanning exons 1–2 of the *Edn1* locus generated a truncated 343‐bp PCR product, confirming successful heterozygous targeting. Homozygous *Edn1* ^−^ *^/^* ^−^ mice die of respiratory failure at birth and therefore do not survive to adulthood. (D) Schematic of choroidal thickness measurements. Five measurement points were placed 1 mm from the optic disc to avoid the physiological increase in choroidal thickness near the disc and were spaced 15° apart to provide a regional assessment of choroidal thickness affected by vortex vein ligation. (E) Bar graphs showing the proportion of eyes with polypoidal lesions detected by ICGA in each subgroup. The numbers of eyes with available data were 28 eyes from 18 mice (28/18), 23/15, 14/14, 27/15, 13/13, and 15/15 for subgroups I–VI, respectively. Between‐group differences were analyzed using the Rao–Scott chi‐square test, accounting for inter‐eye correlation within individual mice, followed by Bonferroni‐adjusted post hoc pairwise comparisons. Groups sharing the same lowercase letter were not significantly different, whereas groups with different lowercase letters were significantly different (*p* < 0.05). The numbers of evaluable eyes and mice, as well as the notation used to indicate statistical significance, also applied to panels (F–H). (F) Box plots showing the number of polypoidal lesions per eye detected by ICGA in each subgroup. Between‐group differences were assessed using GEEs, with individual mice specified as clusters to account for inter‐eye correlation, followed by Bonferroni‐adjusted post hoc pairwise comparisons. (G) Box plots showing the diameters of the thickest vortex vein per eye in each subgroup. Between‐group differences were assessed using GEEs, with individual mice specified as clusters to account for inter‐eye correlation, followed by Bonferroni‐adjusted post hoc pairwise comparisons. (H) Box plots showing mean choroidal thickness in each subgroup. Between‐group differences were assessed using GEEs, with individual mice specified as clusters to account for inter‐eye correlation, followed by Bonferroni‐adjusted post hoc pairwise comparisons. CCH, choroidal circulatory hypertension; EDNRA, endothelin receptor‐A; GEEs, generalized estimating equations.

## DISCUSSION

In this study, we comprehensively delineated the pathogenic mechanisms underlying Pachy PCV through a multiscale framework integrating clinical phenotyping, multi‐omics analyses, murine modeling, and both in vivo and in vitro validation. Using a multicenter PCV dataset, we first proposed a prognosis‐oriented classification, defining pachychoroidal features as key criteria for identifying Pachy PCV, which was associated with poorer responses to anti‐VEGF therapy compared with Non‐pachy PCV. Plasma multi‐omics analyses supported the biological relevance of this classification, revealing distinct molecular signatures in Pachy PCV patients. To mechanistically interrogate these findings, we established a novel Pachy PCV mouse model by inducing CCH and disturbed fluid shear stress via vortex vein ligation. This model successfully recapitulated hallmark pathological features of Pachy PCV. We further revealed that PB‐CNV originated from active PC vein components and identified EDN1 as a central molecular regulator. In vitro experiments confirmed that disturbed fluid shear stress upregulated EDN1 expression in mCECs, and that EDN1/EDNRA signaling drives vasculopathic remodeling and inflammatory activation, promoting pathological angiogenesis. In parallel, systemic *Edn1* haploinsufficiency, pharmacologic inhibition of EDNRA, or mechanical relief of CCH through scleral fenestration each markedly attenuated Pachy PCV formation in vivo. Together, these findings support the hypothesis that CCH acts as a critical pathogenic driver of Pachy PCV, regulating CEC dysfunction and disease progression through the EDN1/EDNRA signaling axis.

The term “pachychoroid” describes an abnormal increase in choroidal thickness. Although the association between a greater SFCT and poor responses to anti‐VEGF therapy has been previously reported [11], SFCT alone might be insufficient to fully represent the pachychoroid phenotype, as it is influenced by multiple factors, including age and axial length [12, 13]. Based on our large‐scale multicenter PCV dataset, we found a constellation of imaging and clinical features, including increased SFCT, the presence of choroidal pachyvessels, a paucity of AMD‐like features, and younger age, that together serve as more comprehensive biomarkers for identifying Pachy PCV. Despite a significant association in multivariable analysis, massive hemorrhage was excluded from the diagnostic criteria. Pathophysiologically, massive hemorrhage is considered a consequence of CCH rather than an intrinsic structural biomarker or an initiating factor. This feature is also highly correlated with other severe complications, such as hemorrhagic retinal detachment, submacular scarring, macular atrophy, and poor visual prognosis [14, 15]. Incorporating massive hemorrhage into the diagnostic criteria would significantly increase the complexity of the classification system and bias it toward advanced disease stages. Furthermore, the primary objective of multivariable regression analysis was to establish baseline diagnostic criteria; thus, post‐treatment variables like injection frequency were intentionally excluded from the criteria formulation. Existing evidence suggested that therapeutic outcomes were driven more by the treatment regimen itself than by the choice of agent [16]. To ensure the statistical robustness of our proposed classification criteria and address the inherent risk of optimism bias when deriving and evaluating criteria within the same cohort, we conducted a rigorous internal validation using the bootstrap resampling method. The validation results demonstrated excellent discrimination and calibration, indicating that the underlying multivariable logistic regression analysis was highly stable. As a result, this classification system not only showed exceptional accuracy in differentiating Pachy PCV from Non‐pachy PCV, but was also supported by proteomic and metabolomic analyses, which revealed distinct molecular signatures in Pachy PCV patients, including differential metabolites such as phytosphingosine and pyruvic acid, underscoring its clinical feasibility and biological validity.

The phenotypic characteristics of Pachy PCV support an independent pathogenic role of CCH, including a greater maximum choroidal vascular diameter ratio, increased curvature of Bruch's membrane toward the choroid within serosanguineous PED, and a markedly higher prevalence of hemorrhagic complications [17, 18]. Histopathological examination of surgically excised PCV specimens from patients also revealed enlarged, dilated venules with intercellular gaps between ECs [19, 20, 21, 22], consistent with choroidal vascular dilation and hyperpermeability secondary to increased hydrostatic pressure. Moreover, recent advances in ultra‐wide‐field ICGA suggest that the CCH could be secondary to the outflow obstruction of the vortex veins [23], a mechanism that has been experimentally validated in several animal models [24, 25, 26]. These observations provide converging evidence that CCH might play a crucial role in the pathogenesis of Pachy PCV. Following previous reports, we surgically ligated vortex veins of the C57BL/6 mouse eyes to simulate CCH. Multi‐modal imaging indeed captured the formation of Pachy PCV‐like lesions. Conversely, simultaneous scleral fenestration to release choroidal hypertension prevented their formation. Fluorescence staining combined with CLSM further confirmed the presence of PB‐CNV within the RPE‐choroidal complex, which closely matched the lesion location in humans. To our knowledge, this represents the first successful induction of polypoidal lesions and BNNs in an animal model driven by the pachychoroidal concept. In contrast, other mouse models based on overexpressing specific genes (e.g., *Fgd*6 and *Htra*1) and with the absence of choroidal changes failed to reflect the choroidal pathophysiology of PCV as a choroidal disease [27, 28, 29, 30]. Our CCH mouse model thereby represents a more pathophysiologically relevant platform for further mechanistic investigations and therapeutic exploration.

Our study further suggested a putative origin of PB‐CNV from active PC vein cells, consistent with previous clinicopathological observations demonstrating that dilated inner choroidal venules, rather than arterioles, correspond to polypoidal structures [31, 32]. Based on the mouse model, the FSS‐AS and HIF‐1 pathways appeared to be important contributors to this process, aligning with human plasma proteomic and metabolomic analyses, in which Pachy PCV patients exhibited elevated levels of FSS‐AS‐associated proteins (e.g., NCF2, PIK3CB, RAC1, and RHOA) and HIF‐1‐related proteins (e.g., CAMK2G, PRKCA, and ENO1). Components of the HIF‐1 pathway likely interact with FSS‐AS signaling as an auxiliary mechanism. For example, CAMK2 has been reported to be upregulated under disturbed shear stress [33], while ENO1 has been implicated in promoting inflammatory responses and mesenchymal transitions in similar hemodynamic contexts [34]. Metabolomic alterations further corroborated these proteomic signatures. These converging lines of evidence underscore the pivotal roles of the two signaling pathways, especially the FSS‐AS pathway, in the pathological changes occurring in the PC vein region. Indeed, FSS is the tangential frictional force exerted by blood flow on ECs and is normally unidirectional and parallel to the vessel wall; disturbances in laminar flow can give rise to disturbed FSS, which is particularly relevant to Pachy PCV. This is supported by recent clinical studies that have documented pulsatile filling of pachyvessels, retrograde flow patterns with bidirectional oscillation, and impaired venous outflow in pachychoroid‐related conditions [31, 32, 35, 36]. Correspondingly, the PC veins are particularly susceptible to disturbed FSS as they are branching vessels located at junctional sites. Disturbed blood flow in these regions may disrupt endothelial homeostasis, induce functional and phenotypic alterations in ECs, and ultimately contribute to the initiation and progression of PCV.

We further revealed EDN1 as a central molecular effector in Pachy PCV, acting at the intersection of the FSS‐AS and HIF‐1 signaling pathways. EDN1 is a 21‐amino‐acid peptide produced by ECs with potent vasoconstrictive effects [37]. Through its interaction with EDNRA, EDN1 regulates cell proliferation, growth, vasomotor tone, and inflammatory responses, while also activating VEGF signaling and promoting angiogenesis [38, 39, 40, 41]. By stimulating the mechanosensitive complexes through OSS, PECAM1 becomes phosphorylated, activating the integrin (ITGAV) and downstream pathways that regulate EDN1 expression, thereby enhancing endothelial migration and angiogenic activity [42, 43, 44, 45] Components of the HIF‐1 pathway, particularly STAT3, can directly bind to the promoter regions of the *EDN1* and *EDNRA* genes, providing an additional transcriptional mechanism linking hypoxic and hemodynamic cues to EDN1 signaling [46]. In our in vivo experiments, vortex vein ligation induced the formation of polypoidal lesions in nearly half of the eyes and significant choroidal thickening. In contrast, *Edn1* haploinsufficiency or pharmacologic inhibition of EDNRA effectively abolished these pathological changes, which directly supported the role of the EDN1/EDNRA axis in the vortex vein ligation‐induced Pachy PCV formation.

By comparing OSS with physiological FSS, together with *Edn1* knockdown and subsequent *Edn1* re‐expression, we demonstrated that OSS upregulates EDN1 expression in mCECs. This was accompanied by marked cytoskeletal reorganization, enhanced cellular motility, and disrupted intercellular junctions. At the molecular level, OSS induced characteristic functional changes, including increased expression of MMP9, IL‐6, c‐Casp3, and ANG‐1, along with decreased expression of the tight junction protein ZO‐1. Furthermore, EDN1 was shown to physically interact with EDNRA, and *Ednra* knockdown largely phenocopied the effects of *Edn1* silencing, indicating that EDNRA is an essential mediator of EDN1 in OSS‐driven changes. Collectively, these findings suggest that, through the EDN1/EDNRA axis, OSS compromises endothelial integrity, weakens cell‐cell adhesion, increases membrane permeability, and promotes endothelial migration, invasion, inflammation, and angiogenesis. These cellular responses to disturbed shear stress might act as the initial phase of vascular dysfunction during the initiation of Pachy PCV.

Several limitations of the study should be acknowledged. First, as our cohort was predominantly Asian, further research is needed to confirm generalizability to other populations. Second, the root of PB‐CNV was inferred via pseudotime trajectory analysis, and definitive evidence would require further experiments such as lineage tracing. In addition, although plasma proteomic and metabolomic analyses consistently demonstrated upregulation of the FSS‐AS and HIF‐1 signaling pathways, significant alterations in circulating EDN1 levels were not detected. This may be attributable to the paracrine nature of EDN1, which is locally produced and rapidly catabolized [47]. This study utilized plasma rather than intraocular fluids due to the invasiveness and complication risks associated with their acquisition. Additionally, anti‑VEGF therapies and limited sample volumes can confound local molecular profiling and hinder multi‑omics scalability. Plasma served as a more feasible and stable substrate for biomarker screening, offering minimal invasiveness and consistency suitable for the outpatient setting. However, we acknowledge that plasma may not fully capture locally acting, rapidly catabolized molecules such as EDN1. To address this, we are establishing a PCV biobank incorporating intraocular fluids to validate these findings in future studies. Third, although a standardized 3 + PRN protocol was strictly implemented, this study was based on a real‐world multicenter clinical cohort. Residual biases and variations in clinical practices across the participating centers, such as the specific number of injections administered during the PRN phase, cannot be entirely eliminated. Additionally, although rigorous internal validation was performed, the proposed classification criteria have not yet been validated in an independent external cohort. Future prospective studies with strictly controlled interventions and external datasets are warranted to further minimize these confounding effects and confirm the broad clinical applicability of this classification system. Fourth, despite successful imaging and characterization of PB‐CNV in the animal model, human PCV specimens were not available for direct comparison. It should be noted that significant anatomical differences exist between mice and humans, particularly the absence of a macula and potential variations in choroidal hemodynamics. This study does not aim to treat PCV solely as a macular disease, since PCV can also occur outside the macular area and even beyond the retinal vascular arcade [48, 49, 50], where dilated choroidal vessels are commonly seen beneath the lesions. This notion is supported by the observation that most polypoidal lesions in our animal model were found around dilated vortex veins. However, in human eyes, the macula is located at the junction of the vortex veins from four quadrants, which may result in more complex alterations in choroidal hemodynamics and fluid shear stress when venous outflow is occluded. In contrast, mouse vortex veins exhibit only unidirectional blood flow, potentially leading to relatively simpler hemodynamic changes. This difference may limit the direct translatability of our model to human pathogenesis. Finally, although EDN1 inhibition effectively suppressed Pachy PCV formation and OSS‐related pathology in preclinical models, further validation in human subjects will be necessary to establish its therapeutic potential.

## CONCLUSION

In conclusion, this study provides multiscale evidence supporting a pathogenic cascade in Pachy PCV, wherein CCH induces disturbed shear stress, activates the FSS‐AS and HIF‐1 signaling pathways, and disrupts CEC homeostasis through the EDN1/EDNRA axis, ultimately promoting pathological angiogenesis and the onset of Pachy PCV. These findings elucidate a previously underappreciated hemodynamic‐molecular mechanism underlying Pachy PCV and highlight potential therapeutic targets for future intervention.

## METHODS

This study received approval from the Institutional Review Board/Ethics Committee of PUMCH (No. I‐25PJ0859, HS‐1538) and was conducted in accordance with the Declaration of Helsinki. Written informed consent was obtained from all included patients. The study's workflow is depicted in Figure S1. The base China map was extracted from the Standard Map Service of the Ministry of Natural Resources of China. Map Approval No. GS(2019)1673.

### Multicenter patient recruitment

Treatment‐naive PCV patients were recruited from Peking Union Medical College Hospital (PUMCH) and 42 other participating centers from May 2017 to May 2023. The inclusion criteria were treatment‐naive PCV patients who received standardized “3 + pro re nata” (3 + PRN) anti‐VEGF regimens and were scheduled for 12 months of follow‐up. The confirmed diagnosis of PCV was based on the EVEREST criteria [51] or the non‐ICGA criteria established by the Asia‐Pacific Ocular Imaging Society PCV Workgroup [2]. The anti‐VEGF agents used included ranibizumab (Lucentis; Genentech), aflibercept (Eylea; Regeneron), and conbercept (KH902; Chengdu Kanghong Biotech Co.), with the first three consecutive injections required to be of the same type. For patients with bilateral PCV, only one eye was included in the study. Exclusion criteria included patients with concurrent chorioretinopathies or ocular inflammatory diseases, previous ocular surgeries (excluding cataract surgery), systemic diseases contraindicating anti‐VEGF therapy, significant refractive media opacities affecting choroid evaluation, or patients with insufficient medical data or lost to follow‐up. Cases without a definitive diagnosis of PCV were also excluded from this study. Patients lost to follow‐up with complete baseline data were excluded from the primary analysis but included in the extreme‐case sensitivity analyses.

### Prognosis‐based classification of Pachy and Non‐Pachy PCV

Fovea‐ and lesion‐centered swept‐source OCT was performed at baseline and during monthly follow‐up visits. Central retinal thickness, SFCT, and maximum PED height were measured three times by two experienced ophthalmologists (X.Y.Z. and W.F.Z.), and the averages were calculated. The presence of AMD‐like features, subretinal fluid (SRF), intraretinal fluid (IRF), massive hemorrhage, and choroidal pachyvessels was independently assessed from OCT images by the same two experienced ophthalmologists (X.Y.Z. and W.F.Z.). Discrepancies were assessed using kappa statistics, and consensus was reached by discussing with the corresponding author (Y.X.C.). AMD‐like features included soft drusen, subretinal drusenoid deposits, characteristic pigment abnormalities, and geographic atrophy [52]. Massive hemorrhage was defined as subretinal or sub‐RPE hemorrhage ≥4‐disc diameters and/or VH [2]. Choroidal pachyvessels were identified as pathological dilation of Haller's layer, accompanied by attenuation and thinning of choriocapillaris and Sattler's layer.

After 12 months of follow‐up, the treatment response to the 3 + PRN anti‐VEGF regimens was classified as either good or poor. Good responders were defined as eyes demonstrating complete resolution of SRF, IRF, and hemorrhage, without PED enlargement, at 12 months after initiating anti‐VEGF therapy. Poor responders were defined as eyes with persistent SRF, IRF, hemorrhage, and/or PED enlargement at the 12‐month follow‐up.

Univariable logistic regression analysis was initially performed, followed by multivariable logistic regression analysis to identify independent variables significantly associated with treatment response, with “poor responder” set as the dependent variable. Factors with *p* < 0.05 in the multivariable analysis were considered as primary criteria, while those with a *p*‐value of greater than 0.05 in multivariable analysis but less than 0.05 in univariable analysis were designated as secondary criteria. To account for within‐center correlation, center‐clustered robust standard errors were used in the multivariable logistic regression analysis. Sensitivity analyses were further conducted using a mixed‐effects multivariable logistic regression model with a random intercept for center and a leave‐one‐center‐out approach to assess the robustness of the findings. Based on the baseline characteristics relevant to treatment prognosis, new‐prognosis‐based classification criteria incorporating pachychoroid features for treatment‐naive PCV were established, dividing PCV patients into Pachy PCV and Non‐pachy PCV. To further verify this classification, explore the potential pathogenic mechanisms, and identify differences in blood biomarkers between these two PCV subtypes, plasma samples were collected from patients in each group according to this classification.

### Internal validation

To evaluate the statistical robustness of the multivariable logistic regression analysis and adjust for potential optimism bias, internal validation was performed using a bootstrap resampling technique with 1000 iterations. The discrimination ability was assessed using the concordance index (C‐index). Furthermore, calibration was evaluated by plotting a calibration curve and calculating the mean absolute error, along with the optimism‐corrected calibration intercept and slope.

### Sample collection for proteomics and metabolomics analysis

Based on the inclusion, exclusion, and diagnostic criteria of the clinical cohort, plasma samples were prospectively and independently collected from 40 Pachy PCV and 31 Non‐pachy PCV patients in the multicenter PCV dataset, along with 35 age‐matched healthy controls from the PUMCH physical examination center. The plasma samples were centrifuged at 1000 × *g* for 10 min and immediately stored at −80°C for preservation.

### Proteomics analysis

Plasma samples were processed using a plasma proteome preparation kit (Cat # PN‐23676). Briefly, samples were centrifuged at 16,000 × *g* for 1 min. Plasma (100 μL) was then mixed with pre‐washed nanoparticles in a centrifuge tube and incubated for 1 h at 37°C with shaking at 1000 rpm. Bound proteins were subsequently washed three times with 500 µL washing buffer for magnetic separation. After washing, 50 µL of lysis buffer was added and incubated at 95°C for 10 min. After cooling to room temperature, the digestion buffer was added, and the samples were incubated at 37°C for enzymatic digestion.

For liquid chromatography‐tandem mass spectrometry (LC‐MS/MS) analysis, an analytical column (Thermo Scientific, 75 μm × 250 mm, 2 μm) on U3000 was connected to an Orbitrap Exploris480 mass spectrometer (Thermo Scientific). A binary solvent system was used for peptide elution: phase A (99.9% H_2_O and 0.1% formic acid) and phase B (80% ACN, 19.9% H_2_O, and 0.1% formic acid), with a linear gradient. The EASY‐Spray ion source operated at a spray voltage of 2.3 kV and a capillary temperature of 320°C. The data‐independent acquisition mass spectrometry method was conducted with a full scan targeting 3e6 ions (350 to 1200 m/z, resolution = 120,000 at 200 m/z), followed by high‐energy collision dissociation of precursor ions across 80 windows (resolution of 30,000). In centroid positive polarity mode, an AGC target of 2e5 ions was set with a maximum injection time of 50 ms.

DIA data were processed using Spectronaut software (version 17.2). Enzyme specificity was set to cleavage at the C‐terminus of arginine and lysine, allowing up to two missed cleavages. Mass tolerance thresholds were set at 10 ppm for initial precursor mass and 0.02 Da for the fragment mass deviation. The FASTA file of human protein data was sourced from Uniprot (2024 release, 20,419 entries). Proteins expressed in over 70% of the samples were included in subsequent analyses. Intergroup comparisons were made using a two‐sided unpaired Welch's *t*‐test, with an adjusted *p* < 0.05 and a fold‐change > 1.3 indicating statistically significant differences in protein expression levels.

### Non‐targeted metabolomics analysis

Plasma samples (100 μL) were mixed with 300 μL of ACN and pre‐cooled at −20°C, then incubated at room temperature after vortexing for 1 min. Processed samples were left overnight, followed by centrifugation at 12,000 rpm (13,800 × *g*, rotor radius = 8.60 cm) and 4°C for 20 min. The supernatant was collected, and its moisture content was adjusted to 50%.

The Acquity UPLC system (Waters Ltd., Elstree, U.K.) with an HSS T3 column (100 mm × 2.1 mm, 1.7 μm; Waters) [53] was utilized for UPLC‐MS analysis in polar ionic mode. Liquid chromatography conditions included phase A (99.9% H_2_O and 0.1% formic acid) and phase B (99.9% ACN and 0.1% formic acid). The column and sample temperatures were set at 40°C and 10°C, respectively, with a flow rate maintained at 0.2 mL/min.

The IDA high‐sensitivity scan mode was employed, incorporating dynamic range reduction for background subtraction. Ion source parameters included cone gas flow (30 L/h), Gas1 flow rate (50 L/h), Gas2 flow rate (50 L/h), temperature (500°C), and scan duration (20 min). Each MS1 scan (60 to 1250 m/z) was followed by 13 MS2 scans (50 to 1250 m/z, accumulation time of 30 ms), with a collision energy set at ±15 eV. For positive ion acquisition, a spray voltage of 5500 V and collision energy of 35 V were applied; for negative ion acquisition, a spray voltage of −4500 V and collision energy of −35 V were used. Quality control samples were inserted every 10 samples to ensure stable sample injection.

### Animal model construction and imaging

Healthy C57BL/6 male mice (~9 weeks, Experimental Animal Research Center, Beijing, China) and heterozygous *Edn1* knockout (*Edn1* ^*+/*^^−^) C57BL/6 mice (~9 weeks, Cyagen Biosciences) were purchased commercially and maintained in a specific‐pathogen‐free mouse facility at the Peking Union Medical College Hospital Animal Care Center. The mice were generated by CRISPR‐Cas9‐mediated conventional (global) knockout involving a 1213‐bp deletion spanning exons 1–2 of the *Edn1* locus. The Edn1^+/−^ genotype was confirmed by PCR genotyping of tail genomic DNA (Figure 7C). Although EDN1 levels were not quantified in isolated choroidal tissue owing to limited protein yield, systemic haploinsufficiency of *Edn1* is known to reduce endothelial endothelin‐1 production across multiple vascular beds, including the ocular circulation [54, 55, 56]. Homozygous *Edn1* ^−^ *^/^* ^−^ mice die of respiratory failure at birth and therefore do not survive to adulthood.

Animal procedures were conducted in accordance with the ARVO Statement on the Use of Animals in Ophthalmic and Vision Research. To investigate the effect of vortex vein ligation, 44 healthy C57BL/6 mice were randomly divided into two groups: control group (*n* = 22) and vortex vein ligation group (*n* = 22). In the latter group, two vortex veins in the superior temporal and superior nasal quadrants of both eyes were surgically ligated using 10‐0 sutures to induce CCH. In the in vivo validation experiments, an additional 30 healthy C57BL/6 mice were randomly divided into the vortex vein ligation combined with scleral fenestration group (*n* = 15) and vortex vein ligation combined with EDNRA inhibitor gavage group (*n* = 15). The same surgical procedure of vortex vein ligation was conducted in the two groups and the *Edn1* ^*+/*^^−^ group (*n* = 15). In the vortex vein ligation combined with EDNRA antagonist gavage group, identical bilateral ligation was performed, followed by daily oral gavage of atrasentan (5 mg/kg; MCE, MedChemExpress) initiated 2 days post‐ligation for 28 consecutive days. The gavage solution was prepared by dissolving 70 mg atrasentan in 7 mL DMSO (MCE, MedChemExpress) to create a 10 mg/mL stock, then daily mixing 10% stock with 40% PEG 300 (MCE, MedChemExpress), 5% Tween‐80 (MCE, MedChemExpress), and 45% physiological saline immediately before administration. For vehicle controls, ligated mice received the same gavage regimen with vehicle solution lacking atrasentan, consisting of 10% DMSO, 40% PEG 300, 5% Tween‐80, and 45% physiological saline. In the vortex vein ligation combined with scleral fenestration group, unilateral ligation was performed in one randomly assigned eye per mouse, with immediate creation of a 0.5‐mm scleral incision 0.5 mm adjacent to ligated veins using a 15° microknife, preserving choroidal integrity to alleviate hypertension. Ligation was also performed on one randomly assigned eye per mouse in the *Edn1* ^*+/*^^−^ group. The control group received sham surgical interventions.

All mice were imaged 30 days after surgery. Mice were anesthetized intraperitoneally with 1.25% Tribromoethanol (Shandong Jitian Biotechnology Co. Ltd.) in 2.8 mL/100 g body weight, and pupils were dilated with 10‐times diluted topical 0.5% tropicamide ophthalmic solution (Santen Pharmaceutical Co. Ltd.). Swept‐source OCT (VG 200 C, Intalight Ltd.) imaging was first performed. Mice were then injected intraperitoneally with a 0.2 mL mixture of 10% sodium fluorescein (Alcon, Alcon Laboratories, Incorporated) and 5 mg/mL indocyanine green (Dalian Beier Pharmaceutical Co. Ltd.) in a 1:3 ratio, followed by simultaneous fundus fluorescein angiography and ICGA (CRO Plus, Microclear Medical Inc.).

One week after imaging, all mice were sacrificed, and the eyes were immediately enucleated. Under a dissecting microscope, the anterior segments, including the cornea, lens, and iris, were removed, and the neurosensory retinas were gently detached from the optic nerve. The remaining eye cups were washed three times in phosphate‐buffered saline (PBS). In the control and vortex vein ligation groups, three mice were allocated for confocal staining and one for H&E staining, while another 18 mice were designated for single‐cell sequencing (6 mice per subgroup, repeated across 3 sets). For confocal staining samples, eight relaxing radial incisions were made, and the remaining RPE‐choroid‐sclera complex was blocked with a buffer containing 1% bovine serum albumin (Sigma, USA) and 0.5% Triton X‐100 (Sigma‐Aldrich) for 60 min at room temperature. This was followed by overnight incubation at 4°C with Isolectin‐B4 (IB4, Invitrogen, Cat. No. I32450) and P‐Selectin (SELP, Proteintech, Cat. No. 13304‐1‐AP). IB4 is an endothelial marker for blood vessels, and SELP, selected according to the specificity and expression rate of differential genes of PB‐CNV found in the scRNA‐seq analysis, is a cell surface glycoprotein that plays a role in vascular inflammation. After washing three times with PBS, secondary antibodies conjugated to FITC‐Labeled Goat anti‐rabbit IgG (1:1000, Servicebio, Cat. No. GB22303) were added and incubated for 60 min to visualize the staining. After three additional washes with PBS, nuclei were counterstained with a ready‐to‐use DAPI staining solution (Servicebio, Cat. No. G1012‐10ML). The slides were then washed three times with PBS and covered with coverslip. Flatmounts were examined and photographed using Leica Stellaris 5 laser scanning confocal microscope (Leica Microsystems, Germany), and the images were analyzed with Las X software. For H&E staining, the enucleated eyes were fixed immediately using 4% formalin and then embedded with paraffin wax. The paraffin‐embedded tissues were sectioned into 4‐μm‐thick sections, and then underwent section mounting, dewaxing through a series of xylene baths, and rehydration through a graded series of ethanol solutions. The sections were then stained with hematoxylin and eosin, covered with a coverslip with a suitable mounting medium to secure the specimen, and then examined under a light microscope.

### ScRNA‐seq analysis

For the 18 C57BL/6 mice in each of the ligation and control groups designated for scRNA‐seq (6 mice per subgroup, repeated across 3 sets), RPE‐choroid‐sclera complexes were harvested and pretreated using the Tissue Dissociation Kit C (SeekMate K01501‐50). After dissociation, samples were incubated in a digestive solution at 37°C until complete digestion or a significant reduction in cell viability was observed. The cell pellet was retained after centrifugation at 300 × *g* and 4°C for 5 min. A Fluorescence Cell Analyzer (Countstar® Rigel S2) was used to assess cell count and viability using an AO/PI reagent. Endothelial cells were enriched prior to single‐cell RNA sequencing for focused analysis. CD45+ cells were magnetically labeled and removed with CD45 MicroBeads (mouse, Miltenyi 130‐052‐301), while CD31+ endothelial cells were collected using MicroBeads (mouse, Miltenyi 130‐097‐418). Of note, this enrichment strategy excludes most immune cells and reduces the recovery of pericytes and stromal cells, limiting comprehensive profiling of the full lesion microenvironment.

ScRNA‐seq libraries were prepared using the SeekOne® Digital Droplet Single Cell 3' Library Preparation Kit (SeekGene Catalog No. K00202). A water‐in‐oil droplet system encapsulated the cells with barcodes and reverse transcription of cDNA, which was quantified via quantitative PCR. High‐throughput paired‐end sequencing was performed on the Illumina platform with a PE150 read length. Read1 contained cell barcodes and unique molecular identifiers (UMI), while Read2 recorded transcriptomic sequences.

The expression matrix of gene‐cell barcodes was constructed using STAR v2.7.10a [57], with further quality control based on UMIs, mitoRatio, and gene expression profiles. Dimensionality reduction, clustering, and visualization were executed using Seurat v4.4.0. Principal component analysis and non‐linear dimensional reduction methods, including uniform manifold approximation and projection (UMAP) and t‐distributed stochastic neighbor embedding [58], were applied to the expression matrix. Dimensionality reduction, clustering, and visualization were performed using UMAP at a resolution of 0.8. A graph‐based method clustered cells, with resolution parameters adjusted to achieve appropriate cell clusters.

### Cell culture and fluid shear stress

mCECs (obtained from Icell, China) were cultured in complete DMEM supplemented with 10% fetal bovine serum (FBS, Atlanta Biologicals) and antibiotics (100 U·mL^−1^ penicillin and 100 μg·mL^−1^ streptomycin; Life Technologies). Cells were maintained at 37°C in a 5% CO_2_ environment.

To model shear stress in vitro, the cells were exposed to stable laminar flow shear stress at 6 dyn/cm^2^ as the control group (CN group), or to OSS at ±6 dyn/cm^2^ as the experimental group (OSS group) for 12 h in complete DMEM using the Fluid Shear Stress system (NK110‐STD, Naturethink). These parameters were selected to accurately recapitulate the pathological hemodynamics of PCV choroidal vasculature: the 6 dyn/cm^2^ baseline aligns with physiological venous/postcapillary shear stress (1–6 dyn/cm^2^) and computational models of human choroidal microvessels [59, 60], while the ±6 dyn/cm^2^ OSS magnitude mirrors the bidirectional/turbulent flow induced by venous obstruction in PCV [25, 61]. The 12 h duration was optimized to reflect chronic low‐grade venous disturbance without cytotoxicity, as lower magnitudes (±2 to ±4 dyn/cm^2^) were biologically inert (Figure S19E,F), whereas higher magnitudes (>10 dyn/cm^2^) compromised cell viability. This regimen falls within the established “pathologically abnormal but non‐cytotoxic” range (4–11 dyn/cm^2^) for vascular endothelium [62]. Approximately 3 × 10^5^ mCECs were seeded on elastin‐coated (12 μg/mL) slides, and glass slides were then placed in a parallel‐plate flow chamber. OSS was calculated by the formula: *τ* = 6 *μQ*/*bh* ^2^, where *τ* is shear stress, *Q* is flow rate, *μ* is medium viscosity, *b* is channel width, and *h* is channel height.

### CCK8 assays, two‐dimensional motility assay, and invasion assay

Cell proliferation was measured using a CCK8 assay, approximately 5000 cells were seeded in 96‐well plates (Corning, Glendale) and incubated with CCK‐8 diluted 1:10 (TargetMol) for 4 h on Day 2. Metabolic activity was measured by optical density at 450 nm using a multi‐well spectrophotometer. A wound‐healing scratch assay was performed to measure cell motility. After seeding cells in 12‐well plates, a scratch was made using a pipette tip in the cell layer. Using an inverted microscope, images of the cell‐free scratch zone were captured at 0 and 48 h post‐scratch. Migration into the scratch zone was quantified using ImageJ software (version 1.54p; National Institutes of Health).

The invasion capacity of cells was assessed using six‐well plates and transwell chambers (Thermo Fisher Scientific) with an 8‐μm pore size. Cell invasion was evaluated in transwell chambers coated with Matrigel (100 μg/mL). A total of 2.5 × 10^5^ cells in 1.5 mL of serum‐free DMEM were placed in the upper chamber, while 2 mL of DMEM‐conditioned medium with 10% FBS was added to the lower chamber. After 48 h, the cells that invaded the lower side of the membrane were stained with Crystal Violet. At least five randomly selected images were taken under a microscope, and the average number of stained cells was counted to represent the relative invasion.

### Real‐time qPCR

An RNeasy Plus mini kit (Qiagen) and a high‐capacity cDNA reverse transcription kit (Applied Biosystems) were utilized to extract total RNA and perform reverse transcription, respectively. Power SYBR green PCR master mix kits (Applied Biosystems) were used to perform real‐time qPCR. Primers are listed in Table S9.

### Western blot analysis

For Western blot, cell lysates were prepared with radioimmunoprecipitation assay buffer supplemented with protease inhibitors (Santa Cruz Biotechnology) and phosphatase inhibitors (Calbiochem). Proteins were separated on 10–15% SDS gels and electrotransferred to polyvinylidene difluoride (PVDF) membranes (Millipore). Membranes were blocked for 1 h using blocking buffer (Bio‐Rad) and subsequently incubated overnight at 4°C with primary antibodies. After washing, membranes were incubated with horseradish peroxidase‐conjugated secondary antibodies (Cell Signaling Technology, anti‐rabbit IgG Cat. No. 7074S or anti‐mouse IgG Cat. No. 7076S) for 45 min at room temperature. Primary antibodies against EDN1 (Abcam, Cat. No. ab117757), EDNRA (Abcam, Cat. No. ab117521), MMP9 (Santa Cruz, Cat. No. sc‐393859), IL‐6 (HUABIO, Hangzhou, HA601051), ZO‐1 (Cell Signaling, Cat. No.13663), c‐Casp3 (Cell Signaling, Cat. No.9661), angiopoietin 1 (HUABIO, Hangzhou, Cat. No. ET1611‐28), and β‐actin (Sigma, Cat. No. A5441) were used. Protein signals were detected using SuperSignal West Femto substrate (Thermo Scientific) and visualized with a luminescent image analyzer (LAS‐3000, Fuji Film) [63].

### Real‐time imaging

A total of 2 × 10^4^ treated cells were uniformly seeded into each well of a six‐well plate to ensure uniform inoculation density. Plates were then placed in an IncuCyte ZOOM live‐cell imaging system (Sartorius), which was set to acquire phase‐contrast images at 60 min intervals over a 24 h period. Image data were exported and further analyzed using ImageJ to quantify cell migration and assess differences between experimental groups.

### Immunoprecipitation

To verify the interaction between EDN1 and its receptor EDNRA, the interaction of EDN1 and EDNRA was examined by co‐immunoprecipitation. Cells were cultured to approximately 80% confluence and then lysed using a lysis buffer (Beyotime) supplemented with protease and phosphatase inhibitors to prevent protein degradation and dephosphorylation. Cell lysates were clarified by centrifugation at 4°C at 10,000 rpm (9570 × *g*, rotor radius = 8.56 cm) for 10 min to remove cellular debris. 35 μL of protein A/G magnetic beads was added to the 400 μL 0.5% PBST; this step was repeated two times. Subsequently, specific EDNRA primary antibodies (Immunoway, Texas, USA) were used. Typically, 10 μg/mL antibody was incubated with the precleared lysates overnight at 4°C with gentle rotation. Unbound proteins were separated using a magnetic rack, and the beads were washed extensively with lysis buffer to remove non‐specific interactions. The immune complexes were then eluted by boiling the beads in 1 × sodium dodecyl sulfate (SDS) sample buffer for 5–10 min. The eluted samples were resolved by SDS‐PAGE and analyzed by Western blot using an EDN1 primary antibody, followed by horseradish peroxidase‐conjugated secondary antibodies for detection.

### Lentiviral transduction of Edn1 and Ednra

To silence the expression of the *Edn1* and *Ednra* genes and overexpress *Edn1* in mCECs, a lentiviral‐mediated silence and stable overexpression transduction approach was employed. mCECs were seeded in a 6‐well plate at a density of 1.2 × 10^5^ cells per well and incubated overnight. When the cells reached 30–50% confluence, 500 μL of fresh culture medium was added to each well. To enhance transduction efficiency, polybrene (0.5 μL) was mixed with lentiviral stocks containing shEdn1 (1.4E8 TU/mL), shEdnra (1.1E8 TU/mL), and *Edn1* lentiviral vectors (3.94E8 TU/mL) separately, and then added dropwise to the cells, followed by incubation in a cell culture incubator for 16 h. After the incubation period, the virus‐containing medium was removed, the cells were washed once with 1 × PBS to eliminate any residual virus, and the PBS was replaced with fresh complete medium, after which cell culturing continued. At 72 h post‐infection, puromycin was added to the culture medium at a concentration of 2.5 μg/mL to select for cells that had integrated the lentiviral constructs. The puromycin selection was maintained for 10 days, with the medium changed every 2–3 days to remove dead cells and replenish puromycin as needed. The knockdown efficiency of the shRNA was validated by assessing mRNA and protein levels using qRT‐PCR and Western blot, respectively.

### Endothelial cell tube formation assay

To assess angiogenic activity in vitro, endothelial cell tube formation assays were performed using Matrigel (Corning). The mCECs were planted on the elastin‐coated slides, and then the shear stress was performed when the confluence reached 80%. Approximately 3 × 10^4^ cells were seeded on the 96‐well plate covered with Matrigel. Tube formation was then visualized under an inverted phase‐contrast microscope (Olympus), and images were captured at 4 × magnification. The extent of tube formation was quantified by measuring the total length of the tubes and the number of branching points using ImageJ software.

### Immunofluorescence

Rhodamine‐conjugated phalloidin was used to visualize F‐actin, and immunofluorescence was conducted to visualize VE‐cadherin. Cell cultures were fixed with 4% paraformaldehyde in PBS for 15 min at room temperature to preserve cellular structures. After fixation, cells were washed three times with PBS to remove residual fixative. Permeabilization was performed using 0.2% Triton X‐100 in PBS for 5 min, and then the cells were washed with PBS again to eliminate the detergent. Non‐specific binding was blocked by incubating the cells with 3% bovine serum albumin (BSA) in PBS for 1 h at room temperature. Primary antibody against VE‐cadherin (Abcam) was diluted in 1% BSA in PBS to a final concentration of 1:100 and applied to the cells, followed by an overnight incubation at 4°C. After incubation with the primary antibody, cells were washed three times with PBS to remove unbound antibodies. A secondary Alexa Fluor 488‐conjugated antibody, specific for the VE‐cadherin, was diluted in 1% BSA in PBS to a final concentration of 1:500 and applied to the cells. The cells were also incubated with a working solution of phalloidin conjugated to rhodamine, diluted in PBS to a final concentration of 1:100, for 1 h at room temperature in the dark. The fluorescent phalloidin binds specifically to F‐actin. After staining, cells were washed with PBS two to three times to remove unbound phalloidin. Finally, the stained cells were analyzed using a fluorescence microscope to capture the actin filaments and VE‐cadherin images.

### Cellular reactive oxygen species detection

According to the manufacturer's instructions, intracellular reactive oxygen species (ROS) levels were evaluated with the Reactive Oxygen Species Assay Kit (Beyotime). Briefly, 5 × 10^4^ cells were seeded in a 96‐well plate for 24 h. Cells were then stained with DCFH‐DA (10 μM) for 30 min, washed three times with serum‐free medium, and obtained for intracellular ROS images under a fluorescence microscope. ImageJ software was used for quantitative analysis of fluorescence intensity; background taken just outside the cells was subtracted from each image.

### Statistical analysis

Continuous variables are presented as mean ± standard deviation (SD) and were analyzed using an independent *t*‐test or a non‐parametric test when data were not normally distributed. Categorical variables are expressed as percentages and were analyzed using the chi‐square test or Fisher's exact test, as appropriate. The sample size, number of poor responders, and poor‐response rate were summarized for each center. Univariable logistic regression analysis was initially performed, followed by multivariable logistic regression analysis with “poor responder” as the dependent variable. Center‐clustered robust standard errors were used in the multivariable analysis to account for potential within‐center correlation. Sensitivity analyses were conducted using a mixed‐effects multivariable logistic regression model with a random intercept for center and a leave‐one‐center‐out approach, in which the multivariable model was refitted after sequentially excluding each of the 43 centers. Furthermore, to assess the potential impact of loss to follow‐up, extreme‐case sensitivity analyses were performed among patients with available baseline data who did not complete the 12‐month follow‐up, assuming that all such patients were either good responders or poor responders. The multivariable logistic regression model with center‐clustered robust standard errors was refitted under both assumptions. R 4.3.2 was used for logistic regression analysis and the bootstrap resampling method. Multiple‐testing correction strategies were applied for omics datasets: permutation‐based FDR for proteomic analysis, Benjamini‐Hochberg FDR for metabolomic analysis, and Bonferroni correction for single‐cell differential expression and pathway enrichment analyses. Differential protein, metabolite, and gene enrichment analyses utilized the clusterProfiler v4.8.3, with GO (org.Hs.eg.db v3.17.0 and org.Mm.eg.db v3.17.0) [64] and KEGG release 111.0 (accessed 2024) [65] databases, and the results were visualized through the DAVID 2021 Update [66] and R version 4.3.1/4.3.2. PPI network was constructed via the STRING v12.0 [67] to analyze protein associations and identify critical pathways, and Cytoscape v3.9.1 [68] with the MCODE algorithm [69] was employed to select hub genes and extract subnetworks to minimize false positives in high‐throughput data. Statistically significant differences in protein, metabolite, and gene expression levels, as well as enriched pathways, were set at *p*‐value < 0.05. Pseudotime trajectory analysis [70, 71, 72] using Monocle2 and Monocle3 algorithms simulated cell developmental trajectories, identifying key branching points and PB‐CNV origins in PCV pathogenesis, with PB‐CNV components selected based on distinct cell clusters, minimal or no presence in controls, and gene expression profiles indicating neovascularization (e.g., energy metabolism, biosynthetic capabilities, or activated cellular states) [73]. In cell‐based experiments, statistical analysis was performed using GraphPad Prism version 11.0.0 (GraphPad Software). Statistical significance was determined using Student's unpaired two‐tailed *t*‐test for two‐group comparisons or one‐way ANOVA followed by Tukey's multiple comparisons test for multiple groups. Data are presented as mean ± SD. For animal experiments, sample sizes were determined to achieve 80% statistical power at a two‐sided significance level of 0.05. Normality was tested with the Shapiro‐Wilk test. In the animal study, because both eyes from some mice were included, analyses accounted for within‐mouse inter‐eye correlation. Categorical outcomes were assessed using the Rao‐Scott chi‐square test, whereas continuous outcomes were analyzed using generalized estimating equations, with individual mice specified as clusters. Bonferroni correction was used to adjust for corresponding post hoc pairwise comparisons. A *p*‐value < 0.05 was considered statistically significant. Single and double asterisks indicated *p‐*value < 0.05 and *p‐*value < 0.01, respectively, in the figures.

## AUTHOR CONTRIBUTIONS

**Xinyu Zhao**: Conceptualization; methodology; data curation; investigation; formal analysis; funding acquisition; project administration; resources; writing—original draft. **Tiantian Cheng**: Conceptualization; methodology; software; investigation; formal analysis; writing—original draft. **Wenfei Zhang**: Conceptualization; investigation; writing—original draft; methodology; data curation; formal analysis. **Xingwang Gu**: Conceptualization; investigation; data curation; formal analysis; writing—review and editing; visualization; writing—original draft. **Qing Zhao**: Data curation; investigation; writing—review and editing; writing—original draft; validation. **Yaning Chen**: investigation; validation; data curation; formal analysis; writing—review and editing. **Lihui Meng**: Data curation; validation; writing—review and editing; software. **Jiaqi Zhang**: Data curation; validation; writing—review and editing; formal analysis. **Bintao Qiu**: Data curation; investigation; writing—review and editing. **Lili Li**: Investigation; data curation; writing—review and editing. **Shiyu Cheng**: Investigation; data curation; writing—review and editing. **Zicheng Wang**: Writing—review and editing; data curation; investigation. **Chuting Wang**: Writing—review and editing; data curation; investigation. **Yuelin Wang**: Writing—review and editing; data curation; investigation. **Zuyi Yang**: Writing—review and editing; data curation; investigation. **Zhengming Shi**: Writing—review and editing; data curation; project administration. **Ye Guo**: Methodology; validation; supervision; project administration; resources; writing—review and editing. **Shengzhi Liu**: Conceptualization; methodology; supervision; formal analysis; funding acquisition; project administration; resources; writing—review and editing. **Youxin Chen**: Conceptualization; validation; supervision; funding acquisition; project administration; resources; writing—review and editing.

## CONFLICT OF INTEREST STATEMENT

The authors declare no conflicts of interest.

## ETHICS STATEMENT

This study received approval from the Institutional Review Board/Ethics Committee of PUMCH (No. I‐25PJ0859 and HS‐1538) and was conducted in accordance with the Declaration of Helsinki. All participants provided written informed consent.

## Supporting information


**Figure S1:** An overall illustration of the technological roadmap and proposed pathogenesis of Pachy PCV.
**Figure S2:** Flow chart of patient enrollment, comparison of baseline imaging parameters between good and poor responders, as well as calibration curve analysis of the established classification system.
**Figure S3:** Supplementary evidence for plasma proteomic and metabolomics analysis among three groups, and multi‐omics intersection exploration.
**Figure S4:** Supplementary result of protein co‐expression analysis in proteomics.
**Figure S5:** Supplementary result of GO enrichment analysis in proteomics.
**Figure S6:** Supplementary results of enrichment analysis in proteomics with MFUZZ algorithm.
**Figure S7:** Supplementary results of proteomic analysis between groups.
**Figure S8:** Disease‐related pathways and proteins in Pachy PCV.
**Figure S9:** Boxplot comparing differential protein expression levels within disease‐related pathways in Pachy PCV in proteomics.
**Figure S10:** Supplementary results of metabolomics analysis between groups.
**Figure S11:** Disease‐related pathways and metabolites in Pachy PCV.
**Figure S12:** Multimodal imaging of the vortex ligation mouse model with classical polypoidal lesions.
**Figure S13:** Fluorescence staining and CLSM of the RPE‐choroid complexes from the vortex ligation mice with polypoidal lesion formation.
**Figure S14:** Supplementary results for cell annotation, assessing the root of PB‐CNV and developmental pathway of neovascularization with pseudotime trajectory analysis.
**Figure S15:** Heatmap about the distribution of differential genes in progenitor cells and subtypes of PB‐CNV.
**Figure S16:** Supplementary results for pseudotime trajectory analysis.
**Figure S17:** Modular genes and enrichment results from pseudotime trajectory analysis.
**Figure S18:** Supplementary results for identifying target genes and associated signaling pathways in Pachy PCV.
**Figure S19:** Effects of proliferation and migration by OSS and shEdn1.
**Figure S20:** Comparison between the vortex ligation, anti‐EDNRA, and vehicle control for anti‐EDNRA groups.

**Table S1:** Baseline characteristics of different response groups.
**Table S2:** Sensitivity analysis using mixed‐effects multivariable logistic regression.
**Table S3:** Sensitivity analysis using leave‐one‐center‐out.
**Table S4:** Distribution of sample sizes across centers.
**Table S5:** Extreme‐case sensitivity analysis for loss to follow‐up.
**Table S6:** Diagnostic performance of the new Pachy PCV criterion against the treatment response.
**Table S7:** List of abbreviation of pathways.
**Table S8:** Cell type and representative annotated genes.
**Table S9:** List of RNA primers.

## Data Availability

Raw data of scRNA‐seq from mouse samples in this study have been deposited in the NCBI Sequence Read Archive under BioProject accession ID: PRJNA1520949 (http://www.ncbi.nlm.nih.gov/bioproject/1520949), with corresponding BioSample accessions SAMN62782526, SAMN62782527, SAMN62782528, SAMN62782529, SAMN62782530, SAMN62782531. Due to ethical and legal restrictions, individual human omics sample data, individual participant data, and the data dictionary cannot be made publicly available. All data are available upon request to the corresponding author and subject to local rules and regulations. The data and scripts used are saved in GitHub https://github.com/TianTianCheng98/Zhao2026/. Supplementary materials (methods, figures, tables, graphical abstract, slides, videos, Chinese translated version, and updated materials) may be found in the online DOI or iMeta Science http://www.imeta.science/.
