# Supplementary material for: Dissecting the angiogenic mechanism of pachychoroid polypoidal choroidal vasculopathy
Source: Imeta. 2026 Sep 30;5(5):e70176. doi: 10.1002/imt2.70176 (PMC13625922; doi:10.1002/imt2.70176)
Supplement: Supplementary file 1 [file IMT2-5-e70176-s001.docx]

**Supporting information to**

**Dissecting the Angiogenic Mechanism of Pachychoroid Polypoidal Choroidal Vasculopathy**

**Running title: Angiogenic Mechanism of Pachychoroid PCV**

Xinyu Zhao^1,2,3#^, Tiantian Cheng^4#^, Wenfei Zhang^1,2,3#^, Xingwang Gu^1,2,3^, Qing Zhao^1,2,3^, Yaning Chen^5,6^, Lihui Meng^1,2,3^, Jiaqi Zhang^1,2,3^, Bintao Qiu^7^, Lili Li^8^, Shiyu Cheng^1,2,3^, Zicheng Wang^5,6^, Chuting Wang^1,2,3^, Yuelin Wang^9^, Zuyi Yang^1,2,3^, Zhengming Shi^1,2,3^, Ye Guo^10*^, Shengzhi Liu^5,6*^, Youxin Chen^1,2,3*^

^1^ Department of Ophthalmology, Peking Union Medical College Hospital, Chinese Academy of Medical Sciences and Peking Union Medical College, Beijing 100730, China.

^2^ Beijing Key Laboratory of Fundus Diseases Intelligent Diagnosis & Drug/Device Development and Translation, Beijing 100730, China.

^3^ Key Laboratory of Ocular Fundus Diseases, Chinese Academy of Medical Sciences, Beijing 100730, China.

^4^ Department of Ophthalmology, the First Affiliated Hospital, School of Medicine, Zhejiang University, Hangzhou, Zhejiang 310003, China.

^5^ School of Pharmaceutical Sciences, Capital Medical University, Beijing 100069, China.

^6^ Beijing Area Major Laboratory of Peptide and Small Molecular Drugs, Capital Medical University, Beijing 100069, China.

^7^ Institute of Clinical Medicine, National Infrastructure for Translational Medicine, Peking Union Medical College Hospital, Chinese Academy of Medical Sciences and Peking Union Medical College, Beijing 100730, China.

^8^ State Key Laboratory of Common Mechanism Research of Major Diseases, Institute of Basic Medical Sciences, Chinese Academy of Medical Sciences and Peking Union Medical College, Beijing 100730, China.

^9^ Department of Ophthalmology, Peking University Third Hospital, Beijing 100096, China.

^10^ Department of Clinical Laboratory, State Key Laboratory of Complex Severe and Rare Diseases, Peking Union Medical College Hospital, Chinese Academy of Medical Science and Peking Union Medical College, Beijing 100730, China.

#These authors contributed equally: Xinyu Zhao, Tiantian Cheng, Wenfei Zhang

* Correspondence: [chenyx@pumch.cn](mailto:chenyx@pumch.cn) (Youxin Chen); [szliu@ccmu.edu.cn](mailto:szliu@ccmu.edu.cn) (Shengzhi Liu); [guoye@pumch.cn](mailto:guoye@pumch.cn) (Ye Guo)

### Figure S1 An overall illustration of the technological roadmap and proposed pathogenesis of Pachy PCV. This study detailly explored the pathogenic mechanism of Pachy PCV.

(A) First, based on a nationwide multicenter patient dataset, we proposed prognosis-based criteria for identifying Pachy PCV. Plasma proteomics and metabolomics analyses of Pachy and Non-pachy patients, as well as normal controls, revealed upregulation of the FSS-AS and HIF-1 signaling pathways specifical to the Pachy PCV group.

(B) We then established a pachychoroid animal model by ligating the vortex veins of C57BL/6 mouse eyes, which induced choroidal thickening and CCH. This model successfully showed the formation of typical PCV-like lesions. During subsequent scRNA-seq analysis of the ocular specimen, pseudotime trajectory analysis indicated that these lesions likely originate from the active PC veins. Gene enrichment analysis again identified the above two pathways (FSS-AS and HIF-1), with EDN1 emerging as a key molecular target. Additional establishment of vortex vein ligation mice with *Edn1* knockout (*Edn1^+/-^*) or EDNRA antagonist validated the role of EDN1 as key player in the development of PCV-like lesions.

(C) Further in vitro experiments using OSS to simulate the impact of CCH on choroidal blood flow validated the pathophysiological effects of OSS on mCECs through the EDN1/EDNRA axis.

(D) Collectively, we identified a novel pathogenic mechanism of Pachy PCV: Vortex vein occlusion induces the CCH status and causes OSS to activate the FSS-AS and HIF-1 pathways in mCECs at the PC venous region via the EDN1/EDNRA axis, which triggers a cascade of downstream events that may contribute to the onset of Pachy PCV.

(E) A corresponding hypothesis on the clinical developing route of human PCV is illustrated.

Pachy = pachychoroid; PCV = polypoidal choroidal vasculopathy; Non-pachy = non-pachychoroid; FSS-AS = fluid shear stress and atherosclerosis; CCH = choroidal circulatory hypertension; scRNA-seq = single-cell RNA-sequencing; PC = posterior-capillary; EDN1 = endothelin-1; EDNRA = endothelin receptor-A; OSS = oscillatory shear stress; mCEC = mouse choroidal endothelial cell; VEGF = vascular endothelial growth factor; CLSM = confocal laser scanning microscopy; RPE = retinal pigment epithelium

### Figure S2 Flow chart of patient enrollment, comparison of baseline imaging parameters between good and poor responders, as well as calibration curve analysis of the established classification system.

(A) Flow chart of patient enrollment.

(B-G) CRT, SFCT, choroidal capillary thickness, choroidal capillary thickness/SFCT, thickness of the Sattler and the Haller layer, and Maximum PED height between good and poor responders.

(H) Calibration curve of the proposed diagnostic criteria for Pachy PCV.

The double asterisks indicate *p* < 0.01.

CRT = central retinal thickness; SFCT = subfoveal choroidal thickness; PED = pigment epithelial detachment

### Figure S3 Supplementary evidence for plasma proteomic and metabolomics analysis among three groups, and multi-omics intersection exploration.

(A) Overlap of differential proteins (left) and metabolites (right) among Pachy PCV, Non-pachy PCV, and control group in the Venn diagram.

(B) Heatmap of protein-metabolite correlation. Pachy PCV vs control group is shown in the left panel, and Pachy PCV vs Non-pachy PCV was shown in the right panel. The single and double asterisks indicate *p* < 0.05 and *p* < 0.01, respectively. The vertical axis contained FSS-AS and HIF-1 pathway related proteins, while the horizontal axis contained FSS-AS, HIF-1, metabolism of energy substances, and stimulus response-related metabolites.

(C) Predictive performance of differential proteins (right) and metabolites (left) in distinguishing Pachy PCV and Non-pachy PCV with the single-variable forecast model.

### Figure S4 Supplementary result of protein co-expression analysis in proteomics. Protein co-expression analysis and GO enrichment results for proteins in each module.

GO = Gene Ontology

### Figure S5 Supplementary result of GO enrichment analysis in proteomics. Grouped bubble plot about GO enrichment analysis of differential proteins among the Pachy PCV, Non-pachy PCV, and control groups.

GO = Gene Ontology

### Figure S6 Supplementary results of enrichment analysis in proteomics with MFUZZ algorithm.

(A) Protein clustering results with MFUZZ algorithm (1, 2, and 3 representing control group, Non-pachy PCV, and Pachy PCV, respectively). Cluster 3, 4, and 7 were dominant in Pachy PCV patients, and their combined enrichment outcomes were presented in (B) KEGG enrichment and GOBP enrichment, respectively.

KEGG = Kyoto Encyclopedia of Genes and Genomes; GOBP = Gene Ontology Biological Process

### Figure S7 Supplementary results of proteomic analysis between groups.

(A) KEGG pathway enrichment analysis of differential proteins between groups with type, specific proteins within each pathway and their classification.

(B) Bar chart about KEGG pathway enrichment analysis of differential proteins between groups.

(C) Heatmap of the correlation of proteins in the same pathway. The four images depict proteins from FSS-AS pathway (Pachy PCV vs control group), HIF-1 pathway (Pachy PCV vs control group), FSS-AS pathways (Pachy PCV vs Non-pachy PCV), and HIF-1 pathway (Pachy PCV vs Non-pachy PCV) , respectively.

### Figure S8 Disease-related pathways and proteins in Pachy PCV.

Heatmap highlighting disease-related pathways and proteins in Pachy PCV.

### Figure S9 Boxplot comparing differential protein expression levels within disease-related pathways in Pachy PCV in proteomics.

### Figure S10 Supplementary results of metabolomics analysis between groups.

(A) PCA plot showing intergroup differences in metabolomics.

(B) Metabolite clustering results with MFUZZ algorithm (1, 2, and 3 representing control group, Non-pachy PCV, and Pachy PCV, respectively).

(C) Bar chart about KEGG pathway enrichment analysis of differential metabolites between groups.

(D) KEGG pathway enrichment analysis of differential metabolites between groups with type, specific metabolites within each pathway and their classification.

PCA = principal component analysis

### Figure S11 Disease-related pathways and metabolites in Pachy PCV.

(A) Heatmap highlighting disease-related pathways and metabolites in Pachy PCV.

(B) Boxplots comparing differential metabolite expression levels with disease-related pathways in Pachy PCV.

### Figure S12 Multimodal imaging of the vortex ligation mouse model with classical polypoidal lesions.

Two vortex veins (blue arrows) were ligated using 10-0 sutures to induce CCH. ICGA showed polypoidal lesions while FFA did not, indicating these lesions derived from the choroidal vessel level.

IR = infrared; RF = red free; FFA = fundus fluorescein angiography; ICGA = indocyanine green angiography

### Figure S13 Fluorescence staining and CLSM of the RPE-choroid complexes from the vortex ligation mice with polypoidal lesion formation.

SELP (green fluorescence) was selected as the biomarker for PB-CNV, with IB4 (red fluorescence) as another biomarker for mCECs. They were used for double mCEC staining of PB-CNV. Blue dashed lines indicate the region of PB-CNV, and yellow arrows indicate polypoidal lesions.

PB-CNV = polypoidal lesion and BNN-related choroidal neovascularization; BNN = branching neovascular network

### Figure S14 Supplementary results for cell annotation, assessing the root of PB-CNV and developmental pathway of neovascularization with pseudotime trajectory analysis.

(A) Bubble plot about gene expression profiles in different cell types.

(B) Differential gene changes along the pseudotime axis (TOP 10). Dot colors correspond to Figure 5A and B.

### Figure S15 Heatmap about the distribution of differential genes in progenitor cells and subtypes of PB-CNV.

Cluster colors correspond to Figure 5A and B.

### Figure S16 Supplementary results for pseudotime trajectory analysis.

Differential gene enrichment results along the developmental path of root-immature PB-CNV-mature PB-CNV. The upper panels represented immature PB-CNV vs root component, while the lower represented mature PB-CNV vs immature PB-CNV. And the upregulated genes were shown on the left while downregulated ones on the right.

### Figure S17 Modular genes and enrichment results from pseudotime trajectory analysis.

### Figure S18 Supplementary results for identifying target genes and associated signaling pathways in Pachy PCV.

(A) GOBP enrichment of upregulated differential genes in active posterior-capillary vein (CNV progenitor cells).

(B) PPI network of upregulated differential genes in CNV progenitor cells displaying specific protein names and associations.

(C) Violin plots comparing differential gene expression levels within FSS-AS and HIF-1 related pathways.

CNV = choroidal neovascularization; PPI = protein-protein interaction

### Figure S19 Effects of proliferation and migration by OSS and shEdn1.

(A) The scratch-based migration of mCECs was promoted by OSS, and the promotion effect was suppressed by *Ednra* shRNA.

(B) Representative images of the cell invasion assay for Figure 6D.

(C) Representative images of the cell migration assay for Figure 6I.

(D) Representative images of the cell invasion assay for Figure 6P.

(E) Decreased proliferation of mCECs by low-velocity OSS and high-velocity OSS.

(F) Effect of scratch-based migration of mCECs by low-velocity OSS and high-velocity OSS.

OSS = oscillatory shear stress (± 6 dyn/cm², 12 h); CN = control (laminar shear stress at 6 dyn/cm²); NC = negative control; shEdn1 = *Edn1* shRNA; oeEdn1 = over-expressed *Edn1*; shEdnra = *Ednra* shRNA

The single and double asterisks indicate *p* < 0.05 and *p* < 0.01, respectively.

### Figure S20 Comparison between the vortex ligation, anti-EDNRA, and vehicle control for anti-EDNRA groups.

(A) Multimodal imaging of a mouse eye in the vehicle control group, where vortex ligation and the solvent for EDNRA antagonist were applied. Polypoidal lesions (yellow arrows) and low-lying BNN-like structures can be seen using CLSM and fluorescence staining.

(B) Comparisons of ocular parameters among the three groups accounted for inter-eye correlation within individual mice. The proportions of eyes with polypoidal lesions were compared among the ligation (23 eyes), vehicle (30 eyes), and EDNRA antagonist gavage (27 eyes) groups using the Rao–Scott chi-square test. Bonferroni-adjusted pairwise comparisons showed that the ligation and vehicle groups had significantly higher proportions of affected eyes than the EDNRA antagonist group (both *p* < 0.05), whereas no significant difference was observed between the ligation and vehicle groups. The number of polypoidal lesions, vortex vein diameter, and choroidal thickness were analyzed using generalized estimating equations. The Bonferroni -adjusted pairwise comparisons showed significantly higher values for all three parameters in the ligation and vehicle groups than in the EDNRA antagonist group (all *p* < 0.05). No significant differences were detected between the ligation and vehicle groups. These findings indicate that the vehicle alone had no detectable effect on the development of PCV-like lesions and support that the observed suppression was attributable to EDNRA antagonism.

The double and triple asterisks indicate *p* < 0.01 and *p* < 0.001, respectively.
